# Utilizing Target Sequences with Multiple Flanking Protospacer Adjacent Motif (PAM) Sites Reduces Off-Target Effects of the Cas9 Enzyme in Pineapple

**DOI:** 10.3390/genes16020217

**Published:** 2025-02-13

**Authors:** Haiyan Shu, Aiping Luan, Hidayat Ullah, Junhu He, You Wang, Chengjie Chen, Qing Wei, Rulin Zhan, Shenghe Chang

**Affiliations:** 1 Tropical Crops Genetic Resources Institute, Chinese Academy of Tropical Agricultural Sciences, Haikou 571101, China; shuhy@catas.cn (H.S.); aipingluan@catas.cn (A.L.); hejunhu123@163.com (J.H.); wangyouid@gmail.com (Y.W.); ccj@catas.cn (C.C.); qingwei_321@163.com (Q.W.); zhanrulin555@163.com (R.Z.); 2 Sanya Research Institute, Chinese Academy of Tropical Agricultural Sciences, Sanya 572025, China; 3 Department of Agriculture, The University of Swabi, Anbar-Swabi 23561, Pakistan; shabkadar@yahoo.com

**Keywords:** protospacer adjacent motif (PAM), CRISPR, Cas9, off target, gRNA

## Abstract

Background/Objectives: CRISPR-Cas9 (Clustered Regularly Interspaced Short Palindromic Repeats)-associated protein 9 is now widely used in agriculture and medicine. Off-target effects can lead to unexpected results that may be harmful, and these effects are a common concern in both research and therapeutic applications. Methods: In this study, using pineapple as the gene-editing material, eighteen target sequences with varying numbers of PAM (Protospacer-Adjacent Motif) sites were used to construct gRNA vectors. Fifty mutant lines were generated for each target sequence, and the off-target rates were counted. Results: Selecting sequences with multiple flanking PAM sites as editing targets resulted in a lower off-target rate compared to those with a single PAM site. Target sequences with two 5′-NGG (“N” represents any nucleobase, followed by two guanine “G”) PAM sites at the 3′ end exhibited greater specificity and a higher probability of binding with the Cas9 protein than those only with one 5′-NGG PAM site at the 3′ end. Conversely, although the target sequence with a 5′-NAG PAM site (where “N” is any nucleobase, followed by adenine “A” and guanine “G”) adjacent and upstream of an NGG PAM site had a lower off-target rate compared to sequences with only an NGG PAM site, their off-target rates were still higher than those of sequences with two adjacent 5′-NAG PAM sites. Among the target sequences of pineapple mutant lines (*AcACS1*, *AcOT5*, *AcCSPE6*, *AcPKG11A*), more deletions than insertions were found. Conclusions: We found that target sequences with multiple flanking PAM sites are more likely to bind with the Cas9 protein and induce mutations. Selecting sequences with multiple flanking PAM sites as editing targets can reduce the off-target effects of the Cas9 enzyme in pineapple. These findings provide a foundation for improving off-target prediction and engineering CRISPR-Cas9 complexes for gene editing.

## 1. Introduction

Inhibiting the expression of a gene is an important method for identifying the gene’s function. RNA interference (RNAi) and creating mutants are two main approaches for inhibiting gene expression [1,2]. RNAi cannot completely knock down the targeted sequence, as it faces unpredictable off-target effects and delivers only temporary or partial inhibition of gene function [3]. Mutants are typically created through EMS induction, T-DNA insertion, or the CRISPR/Cas9 system [4,5,6]. However, the results of EMS induction and T-DNA insertion are random, making it difficult to obtain mutants for specific genes using these methods [4,5]. In contrast, the CRISPR/Cas9 system can introduce mutations in a specific gene within a short time [6]. However, the Cas9 protein may bind to and cut non-target sequences, creating off-target mutations [7]. Such off-target mutations can lead to false phenotypes, which may have downstream effects such as cytotoxicity, genotoxicity, and potential chromosomal rearrangements, ultimately leading to incorrect conclusions about the gene’s function [8]. Notably, the number of off-target mutations is nearly equal to that of on-target mutations in human cells when CRISPR-Cas9 is used [7]. This has significantly hindered the application of CRISPR-Cas9, particularly in clinical studies.

The off-target rate in plants appears to be relatively low. For example, the off-target rate reported in spring barley is 4.2% [9], while in *Arabidopsis*, it is 0.1% [10]. However, another study reported an off-target rate of 2.2% in *Arabidopsis* [11]. In wheat, the off-target rate is 5.7% [12]. Interestingly, using bioinformatic methods, off-target mutations induced by a single guide RNA (sgRNA) in *Arabidopsis* were found to have a specificity score as high as 94, relative to a range of 0–100 [13,14]. In contrast, the off-target rate in the *CPC* gene in *Arabidopsis* was 87% when analyzing mutations in 15 lines [15]. Another study demonstrated the use of CRISPR-Cas9 in *Arabidopsis*, where 14 independent target sequences were successfully edited. Among these, the on-target mutation rates in transgenic plants ranged from 33.7% to 92.3% [16]. Given the potential for false phenotypes and toxic effects on downstream genes, studying methods to reduce the off-target rate in plants is crucial.

Modifying the structure of the Cas9 protein can improve its binding specificity to the target sequence, which will reduce the off-target rate [17]. Cytosine base editors (CBEs) and adenine base editors (ABEs) are two types of CRISPR-based genome editing tools that enable the direct conversion of cytosine to thymine (C-to-T) and adenine to guanine (A-to-G) without inducing double-strand breaks [18,19]. CBEs and ABEs generally have a lower frequency of indel generation compared to traditional CRISPR/Cas9 systems [18,19]. However, the specific frequency of indels can vary depending on the base editor and target sequence [18,19]. Furthermore, these methods are time-consuming and cannot completely eliminate off-target mutations [18,19]. The Cas9 protein has high specificity for 10–12 bp regions upstream of the PAM sequence [20], but its specificity for distal bases is relatively weak [20].

Selecting a specific sequence as an editing target can decrease the off-target rate [7]. However, not all genes contain such sequence fragments. Furthermore, if such sequences are located in the 3′ terminal region of the gene, the gene’s function may not be completely removed, making the results debatable [21]. This could also obstruct the outcomes of subsequent experiments.

After Cas9 binds to the PAM sequence, the Cas9 protein detects and determines whether the sequence upstream of the PAM is the target sequence [22]. If the upstream sequence is not the target, the Cas9 protein moves along the DNA strand to search for the next PAM site [22]. We speculated that if the target sequence has more than one PAM upstream or downstream of the core PAM, the Cas9 protein would require more time to detect the target sequence. This would result in the Cas9 protein lingering in this fragment longer, increasing the likelihood of finding the target sequence and reducing the possibility of off-target mutations. To test this speculation, experiments were conducted in this study.

Additionally, the PAM sequence includes variants such as NAG and NAA [23]. The effects of NAG and NGG have been widely studied in bacteria, yeast, and human cells [11,24,25,26]. However, although NAG and NGG PAM sites have been evaluated and detected in some plants [27], there have been no reports of gene editing in plants using NAG PAM sites to date. In all studies on gene editing in plants, the reported PAM sites have been exclusively NGG [9,15,28]. In this research, besides the NGG PAM, the NAG PAM was also studied, and its effects were compared with those of the NGG PAM. Eighteen target sequences with different numbers of PAM sites were used to construct gRNA vectors. The target sequences from transgenic plants were amplified and sequenced. Experimental results on pineapple confirmed the hypothesis.

## 2. Results

### 2.1. Off-Target Rates in Transformed Pineapples Were Higher with gRNA Vectors Targeting Sequences with One PAM Site Compared to Those with Two or More PAM Sites

To study the effects of multiple PAM sites on gene editing and determine whether multiple PAM sites can reduce the off-target rate, three target sequences (TS1–TS3) containing only a core PAM site and three target sequences (TS4–TS6) with a single flanking PAM site at the 5′ end of the core PAM site were used to construct gRNA vectors (pC1300-Cas9-gRNATS1, pC1300-Cas9-gRNATS2, pC1300-Cas9-gRNATS3, to pC1300-Cas9-gRNATS6, respectively), as described in the materials and methods section. The primers used for constructing gRNA vectors are listed in Table 1, while the primers for amplifying target sequences in transgenic seedlings are provided in Table 2. Target sequences, PAM sites, and gene information are summarized in Table 3.

**Figure 1:**
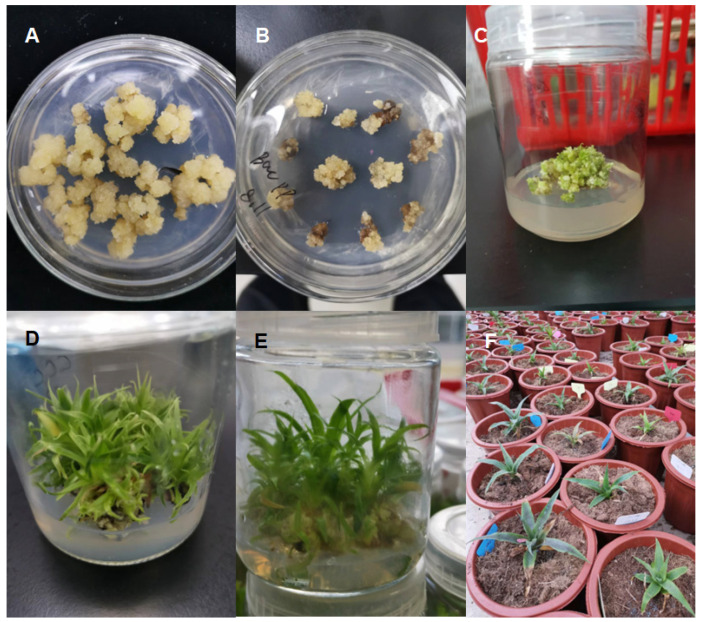
Pineapple callus treated with *Agrobacterium* transformed with the gRNA-AcACS1 vector and regenerated into seedlings. Where (**A**) represents embryogenic callus; (**B**) shows callus infected with *Agrobacterium* and cultured on callus-inducing medium containing 200 mg L^−1^ timentin and 20 µg L^−1^ hygromycin. (**C**–**E**) show callus differentiation into seedlings. (**F**) shows seedlings cultured in pots containing gardening soil.

**Figure 2:**
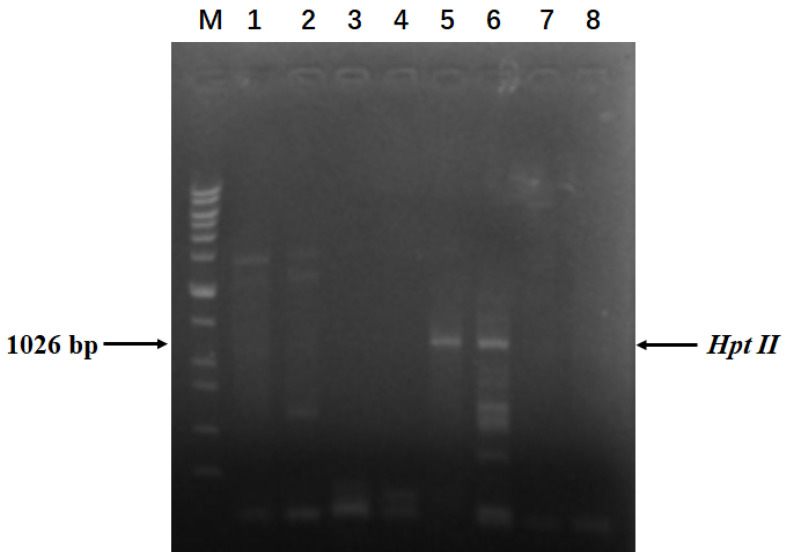
Pinpointing transgenic pineapple plants. Lanes 1-5 represent different plants. PCR products were amplified for the *Hpt II* gene using genomic DNA isolated from different plants as templates. ‘CK’ refers to the non-template control. ‘WT’ refers to the wild-type plant. ‘M’ represents the DNA ladder. The molecular weights of the DNA ladder bands are 20 kb, 15 kb, 10 kb, 7 kb, 5 kb, 3 kb, 2 kb, 1.5 kb, 1 kb, 750 bp, 500 bp, and 200 bp, respectively.

**Figure 3:**
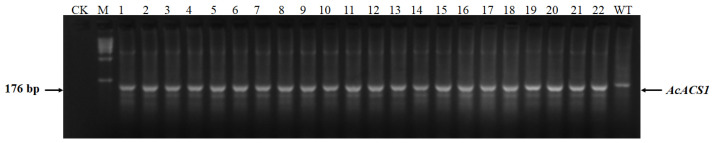
DNA fragments containing target sequences of *AcACS1* amplified using genomic DNA isolated from TS1 mutants as templates. 1–24 represent different TS1 mutant seedlings. ‘CK’ refers to the non-template control. ‘WT’ refers to the wild-type plant. ‘M’ represents the DNA ladder. The molecular weights of the DNA ladder bands are 10 kb, 7 kb, 5 kb, 2 kb, 1 kb, 500 bp, and 200 bp, respectively.

Pineapple embryogenic callus was transformed with *Agrobacterium* carrying the vector pC1300-Cas9-gRNATS1, pC1300-Cas9-gRNATS2, and pC1300-Cas9-gRNATS3 to pC1300-Cas9-gRNATS6, respectively, which were constructed as described in Materials and Methods (Figure 1). Genomic DNA was extracted from the leaves of seedlings regenerated from the transformed callus. The Cas9 vector used in this study [29] harbors a hygromycin-resistance gene (*Hpt II*), which was amplified and sequenced using the extracted genomic DNA as a template (Figure 2). After PCR products were electrophoresed and separated on a 1% agarose gel, plants showing a 1026 bp band were identified as transgenic plants. The DNA fragment containing the target sequence was also amplified using genomic DNA from the transgenic plants as a template and sequenced (Figure 3).

Transgenic plants with edited target sequences were classified as on-target, while those without edited target sequences were considered off-target. Results showed that the off-target rates for TS1–TS3 were 72%, 70%, and 72%, respectively (Table 4 and Tables S1–S3). In contrast, the off-target rates for TS4–TS6 were 48%, 48%, and 46%, respectively (Table 4 and Tables S4–S6). The off-target rates of TS4–TS6 were lower than those of TS1–TS3 (Table 4 and Tables S1–S6), possibly because TS4–TS6 contained one additional PAM site compared to TS1–TS3 (Table 3).

To further investigate this, three target sequences with two flanking PAM sites at the 5′ end of the core PAM sites (TS7–TS9) were also used to construct gRNA vectors (Table 1 and Table 4). Results showed that the off-target rates for TS7–TS9 were 32%, 28%, and 34%, respectively (Table 4 and Tables S7–S9). The off-target rates of TS7–TS9 were lower than those of TS4–TS6 (Table 4 and Tables S4–S9). These results indicate that flanking PAM sites in target sequences can enhance the likelihood of Cas9 binding to the target sequence. Selecting target sequences with flanking PAM sites can effectively decrease the off-target rate in gene-edited plants.

### 2.2. Off-Target Rates in Transformed Pineapples with gRNA Vectors Using Target Sequences with Both 3′ End Flanking PAM Site and Core PAM Site Were Lower than Those with Only a Core PAM Site

Flanking PAM sites in the target sequence can enhance the likelihood of Cas9 binding to the target sequence. Do flanking PAM sites located at the 3′ end of the core PAM site also play a similar role? To address this question, as reported by [15], three sequences (TS10–TS12), each containing a flanking PAM site at their 3′ end (no more than 6 bp from the core PAM site), were used to construct gRNA vectors (Table 1 and Table 4). The off-target rates of plants transformed with gRNA vectors constructed using TS10–TS12 were 48%, 50%, and 48%, respectively (Table 4 and Tables S10–S12). We further confirmed that the off-target rates of TS10–TS12 were less than those of TS1–TS3 (Table 3, Tables S1–S3 and S10–S12) and similar to those of TS4–TS6 (Table 3, Tables S4–S6 and S10–S12). TS1–TS3 did not contain flanking PAM sites, while TS4–TS6 included a flanking PAM site in the target sequence (Table 3) but not at the 3′ end (no more than 6 bp). These results demonstrate that a flanking PAM site at the 3′ end (no more than 6 bp) of the core PAM site can also enhance the possibility of Cas9 binding to the target sequence. In summary, selecting target sequences with a flanking PAM site at the 3′ end can improve the specificity of the Cas9 enzyme.

### 2.3. Off-Target Rates in Transformed Pineapples with gRNA Vectors Using Target Sequences Having NAG Site in the Vicinal Region of the Core PAM Was Less than Those with Only a Core PAM Site

Previous studies suggest that Cas9 can effectively bind to the NAG (“N” represents any nucleobase, followed by adenine “A” and a guanine “G”) site [15,21]. If Cas9 can indeed bind to the NAG site in pineapple cells, then NAG might also enhance the specificity of Cas9. To verify this hypothesis, three sequences with an NAG site and only one PAM (TS13–TS15) were used to construct gRNA vectors (Table 1 and Table 4). The results showed that the off-target rates for TS13–TS15 were 56%, 58%, and 56%, respectively (Table 4 and Tables S13–S15). Additionally, three other sequences, in which an NAG site was located at the 3′ end within 6 bp (TS16–TS18), were selected to construct gRNA vectors (Table 1 and Table 3). In this case, the off-target rates for TS16–TS18 were 58%, 58%, and 56%, respectively (Table 4 and Tables S16–S18). These rates were comparatively lower than those for TS1–TS3 but higher than those for TS4–TS6 (Table 3, Tables S1–S6 and S13–S18). In conclusion, the NAG site near the core PAM can enhance the possibility of Cas9 binding to the target sequence. However, its function was less effective than that of NGG (where “N” represents any nucleobase, followed by two guanines “G”) [30].

### 2.4. Relationship of Mutant Type and Quantity of PAM Sites

The Cas9 enzyme can induce insertions and deletions. Among the target sequences of pineapple mutant lines, more deletions were found than insertions (Table 4). The results indicate that the mutant pineapples exhibited more deletions than insertions (Table 4), suggesting that, after Cas9 binds to the core PAM, deletions are the predominant type of mutations introduced.

## 3. Discussion

CRISPR Cas9 is known for its ability to edit gene sequences and alter nucleotides [31,32,33,34,35]. However, this system often exhibits off-target events, hindering its precise application. Cas9 requires two RNA molecules: a CRISPR RNA (crRNA) and a trans-activating crRNA (tracrRNA), which base-pairs with the repeat sequence of the crRNA [36,37]. The resulting Cas9-RNA complex must first recognize a short DNA sequence motif called the protospacer-adjacent motif (PAM) and then test the flanking double-stranded DNA (dsDNA) for sequence complementarity to the crRNA [38,39]. The crRNA and tracrRNA can be replaced with a simplified, single chimeric single-guide RNA (sgRNA) that is approximately 100 nucleotides in length and fulfills all the functions of the dual crRNA-tracrRNA system found in nature [40]. Similarly, studies suggest that partially replacing RNA nucleotides with DNA nucleotides in crRNA can reduce the off-target rate in human cells. When 10 bases at the 5′ end of VEGFA crRNA were replaced with DNA, the DNA crRNA generated less than 4% off-target indels, whereas the native crRNA produced 10–15% off-target indels [37,40].

Lowering the concentration of gRNA and Cas9 nuclease expressed in cells can reduce off-target events [14]. It has been reported that transfecting lower amounts of sgRNA- and Cas9-expressing plasmids only decreases the mutation rate at the on-target site, without changing the relative rate of off-target mutations [38]. In other words, reducing the expression levels of gRNA and Cas9 in cells does not decrease the off-target rate [38]. Off-target effects during Cas9-mediated genome editing arise from the ability of Cas9 to tolerate mismatches between the gRNA and potential dsDNA targets [41]. Although a single base mismatch within the PAM or seed region can reduce Cas9 cleavage activity, multiple mismatches within the PAM-distal region can be tolerated by Cas9 [42]. When an additional PAM is located 5′ to the core PAM, even if the match between the gRNA and the target sequence is not fully complementary, the gRNA can still bind to a partial target sequence of the dsDNA. This may trigger the HNH-like domain to cleave the target DNA strand upstream of the flanking PAM located 5′ to the core PAM, thereby increasing the off-target rate. Similarly, if another NGG sequence is located 4–6 base pairs (bp) 3′ to the core PAM, the gRNA can bind to a partial target sequence and trigger the HNH-like domain to cleave the target DNA strand upstream of the flanking PAM located 3′ to the core PAM, also increasing the off-target rate.

Cas9 specificity is primarily determined by perfect base pairing within 10–12 bp directly at the 5′ end proximal to the PAM [36,42]. Additionally, sequences 8–12 bp proximal to the PAM, along with PAM-distal sequences, contribute to the overall specificity of Cas9-mediated DNA cleavage [14]. Cas9 interacts weakly with the PAM sequence, yet probes neighboring sequences via facilitated diffusion and can move to another nearby PAM. Consequently, using this interaction to target sequences generates an on-target effect [2]. The Cas9 enzyme may remain in DNA regions containing multiple PAM sites for extended periods. Target sequences containing more PAM sites have an increased potential to bind with the Cas9 enzyme, thereby reducing off-target rates.

The Cas9 protein remains inactive in the absence of gRNA [8,12]. The gRNA is programmed to own a 5′ end, which is complementary to the target sequence. The programmed gRNA binds to the Cas9 and generates changes in the protein, which leads the inactive Cas9 protein into its active form. PAM recognition triggers the initial unwinding of the adjacent dsDNA (referred to as the seed region), allowing the gRNA to undergo strand invasion and form an RNA-DNA hybrid while displacing the non-complementary DNA strand [39]. Once triggered, it searches the target sequence by binding with a sequence that matches the PAM sequence (5′-NGG-3′) [8,12]. Then, Cas9 cuts dsDNA upstream of the PAM [12]. 5′-NAG-3′ and 5′-NGG-3′ are all PAM sites that Cas9 binds and characterizes. But the binding capacity between Cas9 and NAG PAM is weaker than that between Cas9 and NGG PAM [24,30,43,44]. Cas9 dissociates more easily from NAG than from NGG. The possibility that Cas9 finds the core PAM with flanking NAG will be less than that finds the core PAM with flanking NGG. And so, the off-target rate of a core PAM with a flanking NAG was more than that of a core PAM with a flanking NGG.

Our results showed that the off-target rates of TS1–TS3 were 72%, 70%, and 72%, respectively. Furthermore, the off-target rates of TS4–TS6 were 48%, 48%, and 46%, respectively, which were lower than those of TS1–TS3. In Table 4, the average off-target rate for TS4 to TS6 (where an additional NGG is located 5′ to the core PAM) is 47.3%, with a standard deviation of 2.0%. The average off-target rate for TS10 to TS12 (where an additional NGG is located 3′ to the core PAM) is 48.7%, with a standard deviation of 2.0%. The difference between the off-target rates when an additional NGG is located 5′ versus 3′ to the core PAM is not statistically significant. The off-target rates of pineapple plants transformed with gRNA vectors targeting sequences with only one PAM site were higher compared to those with multiple PAM sites. Selecting target sequences containing flanking PAM sites can decrease the off-target rate of gene-edited plants. Additionally, the off-target rate of plants transformed with gRNA vectors constructed using target sequences with flanking PAM sites at the 3′ end and a core PAM site was lower than that of sequences with only a core PAM site. A flanking PAM site at the 3′ end of the core PAM can enhance the likelihood of Cas9 binding to the target sequence. Therefore, selecting target sequences with flanking PAM sites at the 3′ end can improve the specificity of the Cas9 enzyme. Several studies have reported that indels occur 3–4 bp upstream of the PAM sequence [30,31,32]. This result was obtained from experiments with microorganisms and in vitro studies. However, how this occurs in eukaryotes is unknown.

If the DNA sequence is fully complementary to the gRNA, the seed region of the target dsDNA promotes further RNA-DNA hybrid formation, resulting in the formation of a stable R-loop [45]. During R-loop formation, the Cas9 protein undergoes a conformational change from a nuclease-inactive to a nuclease-active state, exposing the target DNA strand to the HNH nuclease active site [46]. In the active conformation, the HNH-like domain cleaves the target DNA strand upstream of the PAM, while the RuvC-like nuclease domain cleaves the non-target strand [46,47]. In contrast to the cleavage of the complementary strand by HNH at the exact -3 position upstream of the PAM, the cleavage of the noncomplementary strand by RuvC is flexible [48]. Specifically, RuvC cleaves the noncomplementary strand at the 3′ end as well as at further upstream positions of the PAM, both in vivo and in vitro, resulting in blunted as well as non-blunted DSB ends with 5′ overhangs [33]. Some studies did not observe this trend at all [49]. Indels occurred randomly in the target sequence in those studies [49]. Similarly, the indels in our results also appeared randomly in the target sequence. The underlying mechanism requires further investigation.

## 4. Conclusions

In this research, using pineapple as the gene-editing material, we found that selecting sequences containing multiple flanking PAM sites as editing targets resulted in a lower off-target rate compared to those with a single PAM site. Target sequences with NGG sites at the 3′ end exhibited greater specificity and a higher probability of binding to the Cas9 protein. NAG plays a similar, but lesser, role compared to NGG. We observed more deletions than insertions in the resultant mutants. These findings could be useful for reducing the off-target rate in future gene-editing experiments.

## 5. Methods

### 5.1. Vector Construction

Forward and reverse primers were synthesized by Sangon Biotech (Shanghai, China). Ten micromoles of forward primers and 10 micromoles of corresponding reverse primers were annealed at 70 °C to synthesize the oligo. Since there was a single Aar I site in the SK-gRNA vector, the synthesized oligo with an Aar I site was directly inserted into the SK-gRNA vector (Table 1) [29]. The resulting plasmid was transformed into Escherichia coli 10B and cultured. The plasmid was extracted and digested with Bgl II and Kpn I. The Cas9 vector used was pC1300-Cas9 [29], which was digested with Kpn I and Bam HI. Since Bgl II and Bam HI are isocaudomers, the SK-gRNA-digested fragment and the pC1300-Cas9-digested fragment can be ligated directly. The resulting fused vectors were named pC1300-Cas9-gRNATS1, pC1300-Cas9-gRNATS1, pC1300-Cas9-gRNATS1, and pC1300-Cas9-gRNATS18, respectively.

### 5.2. Callus Induction

Pineapple callus was induced according to the protocol described in a published paper [50]. Leaves were removed from the crowns of the pineapple cultivar Tainong 17, and the stem was collected. Using a hairbrush and flowing water, soil and pollutant particles were removed from the stem. The cleaned stem was then immersed in tap water for 30 min. Afterward, it was transferred into a container containing tap water and 1% liquid detergent and kept for another 30 min. The stem was subsequently washed with flowing water for 10 min. Next, it was transferred into a 70% ethanol solution for 10 min and placed in a Petri dish inside a super-clean bench. The upper and lower portions of the stem were cut off and discarded. The remaining section was cut into four equal pieces and placed on a callus induction medium (prepared by dissolving 3.4 g Murashige & Skoog Medium (MS) (Shanghai Yuanye Bio-Technology Co., Ltd., Shanghai, China) [51], 1.0 mg 2,4-D, 1.0 mg 6-BA, 1.0 mg NAA, and 7 g agar in 1 L of water). The plates were placed in an incubator at 26 °C. The explants were cultured in the dark for one month. The resulting callus was then transferred to a fresh callus induction medium and propagated for another month.

### 5.3. Pineapple Transformation and Target-Sequence Identification

Pineapple transformation was performed according to published and modified methods [42,52]. The EHA105 strain of *Agrobacterium tumefaciens* was transformed with pC1300-Cas9-gRNA and cultured in YEB (yeast extract broth) liquid medium containing 50 mgL^−1^ kanamycin and 50 mgL^−1^ rifampicin until OD_600_ reached 0.8. The culture was centrifuged at 400 rpm, and the pellet was re-suspended in AAM (Acetosyringone Added Medium) (Shanghai Yuanye Bio-Technology Co., Ltd., Shanghai, China) [51] liquid medium containing 200 µmolL^−1^ acetosyringone, then cultured at 26 °C for 1 h. The embryogenic callus was dipped in the AAM medium for 30 min. After being dried on sterilized filter paper for 1 h, the callus was transferred onto M1 medium (B5 macroelements (Shanghai Yuanye Bio-Technology Co., Ltd.) [53], MS microelements, MS organic substances, MS iron salts, 4% sucrose, 500 mgL^−1^ glutamine, 0.7% agar, 1.0 mgL^−1^ 2,4-D, 1.0 mgL^−1^ KT, 5.0 mgL^−1^ ABA, and 0.1 mgL^−1^ TDZ) and cultured at 26 °C in the dark for 2 consecutive days. The callus was then transferred onto M2 medium (M1, 20 µgL^−1^ hygromycin, 200 mgL^−1^ Timentin) and cultured at 26 °C in the dark for one month. The fresh callus that appeared was transferred onto M3 medium (MS, 6 µmolL^−1^ 6-BA, and 3 µmolL^−1^ NAA) and cultured at 26 °C under a 12 h light/12 h dark photoperiod for 1 month, where it differentiated into seedlings. Primers P1F: 5′-CTATTTCTTTGCCCTCGGACGAGTG-3′; P1R: 5′-ATGAAAAAGCCTGAACTCACCGCGA-3′ were designed based on the *Hpt II* gene in the plasmid pC1300-Cas9 (GenBank accession number AF234296) [19]. Genomic DNA was extracted from the leaves of potential transgenic plants using the Genomic DNA Extraction Kit (Thermo Fisher, Waltham, MA, USA), following the manufacturer’s instructions. A total of 100 ng of gDNA was used as a template in the PCR reaction. The PCR experiment was conducted using the following protocol: initial denaturation at 94 °C for 4 min; followed by 40 cycles of 94 °C for 30 s, 58 °C for 30 s, 72 °C for 1 min; and a final extension at 72 °C for 10 min. The PCR instrument used in this study was the Prism^®^ 7000 (Applied Biosystems, Foster City, CA, USA). PCR products were analyzed on a 1% agarose gel containing 0.2% Gold View (Aladdin Reagent Co., Ltd., Shanghai, China). The predicted molecular weight of the PCR product was 1026 bp. Upon observation of a band corresponding to 1 kb, the fragment was recovered using a DNA Column Recovery Kit (Beijing Biolab Company, Beijing, China), following the manufacturer’s protocol. The recovered DNA fragment was sequenced by Sangon Biotech (Shanghai, China). If the sequence matched that of the hygromycin phosphotransferase *Hpt II* gene, the seedling was considered transgenic. Genomic DNA was then extracted from the leaves of the transgenic seedlings. Primers were designed based on the detailed information provided in Table 2. PCR was performed using the same protocol as described above. The amplified DNA fragment was purified and sequenced.

Previous research suggests that 10–12 nucleotide bases at the 5′ end of the PAM site directly determine the specificity of Cas9 [14]. A BLAST search was performed on the pineapple genome using the 10 bp nucleotide sequence immediately upstream of the PAM site as a query. If the resulting sequence contained an identical 10 bp sequence to the target sequence but differed in other regions, it was considered a similar target sequence.

After gene editing, genomic DNA was extracted from the leaves of the transgenic plants. Both the target sequence and similar target sequences were sequenced. If the target sequence was identical to that of the wild type, the plant was considered off-target. If the target sequence differed from the wild type and at least one similar target sequence was also different from the wild type, the plant was also considered off-target. A plant was considered on-target only when the target sequence differed from the wild type, but all similar sequences remained identical to those of the wild type.

## Figures and Tables

**Table 1:** Primers used for synthesizing oligos in constructing gRNA vectors.

Target Seq.	Forward Primers (5′ to 3′)	Reverse Primers (5′ to 3′)
TS1	GGCAgcgttttgtctcgctgaccc	AAACgggtcagcgagacaaaacgc
TS2	GGCAtcgtgaaattcgtaaacgaa	AAACttcgtttacgaatttcacga
TS3	GGCAgaacatcattaccatcgtaa	AAACttacgatggtaatgatgttc
TS4	GGCAgcaatttgtaacgtgatggt	AAACaccatcacgttacaaattgc
TS5	GGCAgatcgattattgcgctgtgg	AAACccacagcgcaataatcgatc
TS6	GGCAgttattgacggttaatgtgc	AAACgcacattaaccgtcaataac
TS7	GGCAgtggatcaagaatcacccgg	AAACccgggtgattcttgatccac
TS8	GGCAtcaaaggacttcggcctccc	AAACgggaggccgaagtcctttga
TS9	GGCAagaggctgtcggagaggcac	AAACgtgcctctccgacagcctct
TS10	GGCAatcgccaccaacggccgcca	AAACtggcggccgttggtggcgat
TS11	GGCAgcgcgaagaggctgtcggag	AAACctccgacagcctcttcgcgc
TS12	GGCAattataggttacttgtaccc	AAACgggtacaagtaacctataat
TS13	GGCAgtcaagctcaatgtgtcccc	AAACggggacacattgagcttgac
TS14	GGCAcaccgacgacccgaagcaac	AAACgttgcttcgggtcgtcggtg
TS15	GGCAtccacgtgccgttcacgagc	AAACgctcgtgaacggcacgtgga
TS16	GGCAtcctgacccctattgtttac	AAACgtaaacaataggggtcagga
TS17	GGCAatcttctttgctttctctta	AAACtaagagaaagcaaagaagat
TS18	GGCAcgatccgaattcgccgaaag	AAACctttcggcgaattcggatcg

**Table 2:** Primers used for amplifying target sequences in transgenic seedlings.

Target Seq.	Forward Primers (5′ to 3′)	Reverse Primers (5′ to 3′)
TS1	tattttaatggacatgtcgcattcg	gaaccttttgatatcccagatgcct
TS2	acagatttaatcgcgatctccggtg	gcgcaactaaccaccgtgtcgttgt
TS3	atttcgatatctcccgtgtgtgctc	tgcatctgtttctcttctttcaatt
TS4	ttcatgacggtcttcgtgacgagct	tactgagatttatcttactataatc
TS5	tcttctttgctttctcttatggcct	gattccccaccagggaagctgcagc
TS6	gtgaagctgtaaatatagaacttaa	actagactgtaaatgtgcctagcca
TS7	cgtaatccaaatgggactctccgag	tttaattcaagttaagagtaaagta
TS8	taaaaggagtactgataaccaaccc	gagaagatcctgtgcctctccgaca
TS9	tccacattgcgtacagtctctcaaa	gcgatgcagaccctgaaccaacctg
TS10	tattcatacgcatttcaacgtccag	tagtcttatatatacacaagaatga
TS11	tccacattgcgtacagtctctcaaa	gcgatgcagaccctgaaccaacctg
TS12	ccttcatattcacgctgccgatcgc	ccgtcgtcagcagccaccacgccgt
TS13	acctactgggcgccatgctatccga	aaatctattctcaagaccgaattat
TS14	catgtattgtgatccatatacagca	acggcacgtggatgggtgcggtcgg
TS15	gagcaagtccggctcacggtcccga	agaacatagatcatttatatacctg
TS16	tcccttcaataaccgcgctatcttt	gaaagagtagccgtgagagtcgcga
TS17	tgatggtaggctttgctctcattgt	acggtcgttttccgccacagcgcaa
TS18	gacgtgcgatcttctggcggttcga	agccgagcttctttagcagcctgaa

**Table 3:** Target sequences, PAM sites, and gene information.

Target Seq.	Gene Name	Sequence	Definition	GenBank Accession
TS1	*AcACS1*	gcgttttgtctcgctgacccTGG	1-aminocyclopropane-1-carboxylate synthase-like	NC_033623.1
TS2	*AcACS1*	tcgtgaaattcgtaaacgaaAGG	1-aminocyclopropane-1-carboxylate synthase-like	NC_033623.1
TS3	*AcOT5*	gaacatcattaccatcgtaaAGG	Oligopeptide transporter 5	LSRQ01000073.1
TS4	*AcOT5*	gcaatttgtaacgtgatggtAGG	Oligopeptide transporter 5	LSRQ01000073.1
TS5	*AcOT5*	gatcgattattgcgctgtggCGG	Oligopeptide transporter 5	LSRQ01000073.1
TS6	*AcCSPE6*	gttattgacggttaatgtgcTGG	Cellulose synthase-like protein E6	LSRQ01000073.1
TS7	*AcACS1*	gtggatcaagaatcacccggAGG	1-aminocyclopropane-1-carboxylate synthase-like	NC_033623.1
TS8	*AcACS1*	tcaaaggacttcggcctcccCGG	1-aminocyclopropane-1-carboxylate synthase-like	NC_033623.1
TS9	*AcACS1*	agagcctgtcggagaggcacAGG	1-aminocyclopropane-1-carboxylate synthase-like	NC_033623.1
TS10	*AcACS1*	atcgccaccaacggccgccaCGGcgagg	1-aminocyclopropane-1-carboxylate synthase-like	NC_033623.1
TS11	*AcACS1*	gcgcgaagaggctgtcggagAGGcacagg	1-aminocyclopropane-1-carboxylate synthase-like	NC_033623.1
TS12	*AcOT5*	attataggttacttgtacccTGGtagg	Oligopeptide transporter 5	LSRQ01000073.1
TS13	*AcACS1*	gtc**aag**ctcaatgtgtccccCGG	1-aminocyclopropane-1-carboxylate synthase-like	NC_033623.1
TS14	*AcOT5*	caccgacgacccg**aag**caacCGG	Oligopeptide transporter 5	LSRQ01000073.1
TS15	*AcOT5*	tccacgtgccgttcac**gag**cTGG	Oligopeptide transporter 5	LSRQ01000073.1
TS16	*AcOT5*	tcctgacccctattgtttacTGG**aag**	Oligopeptide transporter 5	LSRQ01000073.1
TS17	*AcOT5*	atcttctttgctttctcttaTGGcct**gag**	Oligopeptide transporter 5	LSRQ01000073.1
TS18	*AcPKG11A*	cgatccgaattcgccgaaagCGGa**aag**	Protein kinase G11A	LSRQ01000073.1

Note: Core PAM sites are shown in capital black letters. Flanking NGG sites are shown in red lowercase letters. Whereas, flanking NAG sites are shown in bold blue lowercase letters.

**Table 4:** Off-target rates and mutation type events.

Target Seq.	Off-Target Rate (%)	On-Target Rate (%)	Deletions	Insertions
TS1	72	28	87	45
TS2	70	30	93	61
TS3	72	28	93	71
TS4	48	52	115	66
TS5	48	52	95	55
TS6	46	54	103	60
TS7	32	68	107	65
TS8	28	72	108	79
TS9	34	66	86	56
TS10	48	52	79	63
TS11	50	50	86	62
TS12	48	52	94	60
TS13	56	44	83	55
TS14	58	42	86	57
TS15	56	44	92	54
TS16	58	42	82	55
TS17	58	42	93	59
TS18	56	44	92	54

## Data Availability

All data are hereby available in the manuscript. Materials used in the study are freely available.

## Supplementary Materials

The following supporting information can be downloaded at: https://www.mdpi.com/article/10.3390/genes16020217/s1. Table S1A. Target sequence of TS1 mutant lines. Table S1B. Off-target sites of TS1. Table S2A. Target sequence of TS2 mutant lines. Table S2B. Off target sites of TS2. Table S3A. Target sequence of TS3 mutant lines. Table S3B. Off target sites of TS3. Table S4A. Target sequence of TS4 mutant lines. Table S4B. Off target sites of TS4. Table S5A. Target sequence of TS5 mutant lines. Table S5B. Off target sites of TS5. Table S6A. Target sequence of TS6 mutant lines. Table S6B. Off target sites of TS6. Table S7A. Target sequence of TS7 mutant lines. Table S7B. Off target sites of TS7. Table S8A. Target sequence of TS8 mutant lines. Table S8B. Off target sites of TS8. Table S9A. Target sequence of TS9 mutant lines. Table S9B. Off target sites of TS9. Table S10A. Target sequence of TS10 mutant lines. Table S10B. Off target sites of TS10. Table S11A. Target sequence of TS11 mutant lines. Table S11B. Off target sites of TS11. Table S12A. Target sequence of TS12 mutant lines. Table S12B. Off target sites of TS12. Table S13A. Target sequence of TS13 mutant lines. Table S13B. Off target sites of TS13. Table S14A. Target sequence of TS14 mutant lines. Table S14B. Off target sites of TS14. Table S15A. Target sequence of TS15 mutant lines. Table S15B. Off target sites of TS15. Table S16A. Target sequence of TS16 mutant lines. Table S16B. Off target sites of TS16. Table S17A. Target sequence of TS17 mutant lines. Table S17B. Off target sites of TS17. Table S18A. Target sequence of TS18 mutant lines. Table S18B. Off target sites of TS18.
