# Supplementary material for: Utilizing Target Sequences with Multiple Flanking Protospacer Adjacent Motif (PAM) Sites Reduces Off-Target Effects of the Cas9 Enzyme in Pineapple
Source: Genes (Basel). 2025 Feb 13;16(2):217. doi: 10.3390/genes16020217 (PMC11855603; doi:10.3390/genes16020217)
Supplement: Supplementary file 1 [file genes-16-00217-s001.zip › genes-3414365-supplementary.pdf]

Supplementary File

Table S1A. Target sequence of TS1 mutant lines. Indels are shown in red capital letters.

Line	Target sequence	Deletion	Insertion
Control	GCGTTTTGTCTCGCTGACCC		
TS1-1	GCGTTTTGTCTCGGTGACCC	C14	G14
TS1-2	GCGTTTTGTCTCGCTACCC	G16	
TS1-3	GCGTTTTGTCTCGCTTCCC	G16A17	T16
TS1-4	GCGTTTTGTCTCGCTTACCC	G16	T16
TS1-5	GCGTTTTGTCTCGCAACCC	T15G16	A15
TS1-6	GCGTTTTGTCTCGCTGCCC	A17	
TS1-7	GCGTTTTGTCTCGCCCC	T15G16A17	
TS1-8	GCGTTTTGTCTCGCTAACCC	G16	A16
TS1-9	GCGTTTTGTCTCGCTGTCCC	A17	T17
TS1-10	GCGTTTTGTCTCGCACCC	T15G16	
TS1-11	GCGTTTTGTCTCGCTCCC	G16A17	
TS1-12	GCGTTTTGTCTCGCTGGCCC	A17	G17
TS1-13	GCGTTTTGTCTCGCTCACCC	G16	C16
TS1-14	GCGTTTTGTCTCGCTCCCC	G16A17	C16

Table S1B. Off-target sites of TS1. Indels are shown in red capital letters. Whereas, PAM sites are shown in blue capital letters.

Line	Target sequence	Deletion	Insertion	Position
OT1-1	TCGCTGACCC**AGG**			
OS1-1	TCGCTGCCC**AGG**	A7		LG15 1618630-1618618
OS1-2	TCGCTCACCC**AGG**	G6	C6	LG15 1618630-1618618
OS1-3	TCGCTCCC**AGG**	G6A7		LG15 1618630-1618618
OS1-4	TCGCCAACCC**AGG**	T5G6	C5A6	LG15 1618630-1618618
OS1-5	TCGAACCC**AGG**	C4T5G6	A4	LG18 2055560-2055548
OS1-6	TCGCTTACCC**AGG**	G6	T6	LG18 2055560-2055548
OS1-7	TCGCTCTCCC**AGG**	G6A7	C6T7	LG18 2055560-2055548
OS1-8	TCGCTGGCCC**AGG**	A7	G7	LG18 2055560-2055548
OS1-9	TCGCCCTCCC**AGG**	T5G6A7	C5C6T7	LG20 8211665-8211677
OS1-10	TCGCACCC**AGG**	T5G6		LG20 8211665-8211677
OT1-2	TCGCTGACCC**TGG**			
OS1-11	TCGCTCCCCC**TGG**	G6A7	C6C7	LG4 14670574-14670586
OS1-12	TCGCTAACCC**TGG**	G6	A6	LG4 14670574-14670586
OS1-13	TCGCTTCCC**TGG**	G6A7	T6	LG4 14670574-14670586
OS1-14	TCGCTGCCCC**TGG**	A7	C7	LG4 14670574-14670586
OS1-15	TCGCCCCCC**TGG**	T5G6A7	C5C6	LG4 14670574-14670586
OS1-16	TCGCCAACCC**TGG**	T5G6	C5A6	LG6 9228550-9228562
OS1-17	TCGCTACCC**TGG**	G6		LG6 9228550-9228562
OS1-18	TCGATACCC**TGG**	C4T5G6	A4T5	LG6 9228550-9228562
OS1-19	TCGCTTTCCC**TGG**	G6A7	T6T7	LG6 9228550-9228562

OT1-3	TCGCTGACCC**CGG**			
OS1-20	TCGACCC**CGG**	C4T5G6		LG13 7263449-7263437
OS1-21	TCGCCCC**CGG**	T5G6A7		LG13 7263449-7263437
OS1-22	TCGCT**A**ACCC**CGG**	G6	A6	LG13 7263449-7263437
OS1-23	TCGC**CC**ACCC**CGG**	T5G6	C5C6	LG13 7263449-7263437
OS1-24	TCGCT**CT**CCC**CGG**	G6A7	C6T7	LG13 7263449-7263437
OT-4	TCGCTGACCC**GGG**			
OS1-25	TCGCTG**T**CCC**GGG**	A7	T7	LG6 8570478-8570466
OS1-26	TCG**AA**ACCC**GGG**	C4T5G6	A4A5	LG6 8570478-8570466
OS1-27	TCGCT**TT**CCC**GGG**	G6A7	T6T7	LG6 8570478-8570466
OS1-28	TCGCT**C**ACCC**GGG**	G6	C6	LG6 8570478-8570466
OS1-29	TCGC**AC**ACCC**GGG**	T5G6	A5C6	LG6 8570478-8570466
OS1-30	TCGCTG**C**CCC**GGG**	A7	C7	LG6 8570478-8570466
OS1-31	TCGCT**TT**CCC**GGG**	G6A7	T6T7	LG6 8570478-8570466
OS1-32	TCGC**AT**ACCC**GGG**	T5G6	A5T6	LG6 8570478-8570466
OS1-33	TCGCTG**G**CCC**GGG**	A7	G7	LG6 8570478-8570466
OS1-34	TCGCT**TG**CCC**GGG**	G6A7	T6G7	LG6 8570478-8570466
OS1-35	TCGCTG**G**CCC**GGG**	A7	G7	LG6 8570478-8570466
OS1-36	TCGCT**TG**CCC**GGG**	G6A7	T6G7	LG6 8570478-8570466

Table S2A. Target sequence of TS2 mutant lines. Indels are shown in red capital letters.

**Line**	**Target sequence**	**Deletion**	**Insertion**
Control	TCGTGAAATTCGTAAACGAA		
TS2-1	TCGTGAAATTCGTAAAGAA	C17	
TS2-2	TCGTGAAATTCGTAA**T**CGAA	A16	T16
TS2-3	TCGTGAAATTCGTAA**G**GAA	A16C17	G16
TS2-4	TCGTGAAATTCGTACGAA	A15A16	
TS2-5	TCGTGAAATTCGTAAA**A**GAA	C17	A17
TS2-6	TCGTGAAATTCGTAGAA	A15A16C17	
TS2-7	TCGTGAAATTCGTAAGAA	A16C17	
TS2-8	TCGTGAAATTCGTAACGAA	A16	
TS2-9	TCGTGAAATTCGTA**G**GAA	A15A16C17	G15
TS2-10	TCGTGAAATTCGTA**C**CGAA	A15A16	C15
TS2-11	TCGTGAAATTCGTAAA**G**GAA	C17	G17
TS2-12	TCGTGAAATTCGTCGAA	A14A15A16	
TS2-13	TCGTGAAATTCGTAA**T**GAA	A16C17	T16
TS2-14	TCGTGAAATTCGTAA**C**CGAA	A16	C16
TS2-15	TCGTGAAATTCGTAAA**AA**GAA	C17	A17A18

Table S2B. Off target sites of TS2. Indels are shown in red capital letters. Whereas, PAM sites are shown in blue capital letters.

Line	Target sequence	Deletion	Insertion	Position
OT2-1	CGTAAACGAAAGG			
OS2-1	CGTAAAGAAAGG	C7		LG5: 8196468-8196480
OS2-2	CGTAAGCGAAAGG	A6	G6	LG5: 8196468-8196480
OS2-3	CGTACTCGAAAGG	A5A6	C5T6	LG7: 6693072-6693084
OS2-4	CGTCTGCGAAAGG	A4A5A6	C4T5G6	LG7: 6693072-6693084
OS2-5	CGTATTGGAAAGG	A5A6C7	T5T6G7	LG7: 6693072-6693084
OS2-6	CGTAAGAAAGG	A6C7		LG10: 5541695-5541707
OS2-7	CGTAATCGAAAGG	A6	T6	LG10: 5541695-5541707
OS2-8	CGTAAAAGAAAGG	C7	A7	LG10: 5541695-5541707
OS2-9	CGTCGAAAGG	A4A5A6		LG23: 6060177-6060165
OS2-10	CGTAAGTGAAAGG	A6C7	G6T7	LG23: 6060177-6060165
OS2-11	CGTAACCGAAAGG	A6	C6	LG23: 6060177-6060165
OT2-2	CGTAAACGAAGGG			
OS2-12	CGTAGGGAAGGG	A5A6C7	G5G6	LG1: 11738825-11738837
OS2-13	CGTAAGGGAAGGG	A6C7	G6G7	LG1: 11738825-11738837
OS2-14	CGTAACGAAGGG	A6		LG1: 11738825-11738837
OS2-15	CGTCGTCGAAGGG	A4A5A6	C4G5T6	LG5: 11745654-11745666
OS2-16	CGTAAATGAAGGG	C7	T7	LG5: 11745654-11745666
OS2-17	CGTAGCCGAAGGG	A5A6	G5C6	LG5: 11745654-11745666
OS2-18	CGGGCCGAAGGG	T3A4A5A6	G3G4C5	LG6: 8721179-8721167
OS2-19	CGTAAGAAGGG	A6C7		LG6: 8721179-8721167
OS2-20	CGTAATCGAAGGG	A6	T6	LG14: 6222275-6222263
OS2-21	CGTGCCGAAGGG	A4A5A6	G4C5	LG14: 6222275-6222263
OS2-22	CGTAAAAGAAGGG	C7	A7	LG14: 6222275-6222263
OT2-3	CGTAAACGAACGG			
OS2-23	CGTTGTGAACGG	A4A5A6C7	T4G5T6	LG4: 11629679-11629667
OS2-24	CGTAATTGAACGG	A6C7	T6T7	LG4: 11629679-11629667
OS2-25	CGTACCGAACGG	A5A6	C5	LG4: 11629679-11629667
OS2-26	CGTAAGCGAACGG	A6	G6	LG4: 11629679-11629667
OS2-27	CGTTCGAACGG	A4A5A6	T4	LG4: 11629679-11629667
OS2-28	CGTAAATGAACGG	C7	T7	LG25: 2959978-2959990
OS2-29	CGTATTCGAACGG	A5A6	T5T6	LG25: 2959978-2959990
OS2-30	CGTAAGTGAACGG	A6C7	G6T7	LG25: 2959978-2959990
OS2-31	CGTAACCGAACGG	A6	C6	LG25: 2959978-2959990
OS2-32	CGTAATTGAACGG	A6C7	T6T7	LG4: 11629679-11629667
OS2-33	CGTATTCGAACGG	A5A6	T5T6	LG25: 2959978-2959990
OS2-34	CGTAAGTGAACGG	A6C7	G6T7	LG25: 2959978-2959990
OS2-35	CGTTCGAACGG	A4A5A6	T4	LG4: 11629679-11629667

Table S3A. Target sequence of TS3 mutant lines. Indels are shown in red capital letters.

Line	Target sequence	Deletion	Insertion
Control	GAACATCATTACCATCGTAA	/	/
TS3-1	GAACATCATTACCATCTAA	G17	
TS3-2	GAACATCATTACCATAGTAA	C16	A16
TS3-3	GAACATCATTACCATTTAA	C16G17	T16
TS3-4	GAACATCATTACCAGTAA	T15C16	
TS3-5	GAACATCATTACCATCCTAA	G17	C17
TS3-6	GAACATCATTACCGTAA	A14T15C16	
TS3-7	GAACATCATTACCATTTAA	C16G17	T16
TS3-8	GAACATCATTACCATTGTAA	C16	T16
TS3-9	GAACATCATTACCGGAGTAA	A14T15C16	G14G15A16
TS3-10	GAACATCATTACCATCTTAA	G17	T17
TS3-11	GAACATCATTACCAGAGTAA	T15C16	G15A16
TS3-12	GAACATCATTACCATCATAA	G17	A17
TS3-13	GAACATCATTACCAAAGTAA	T15C16	A15A16
TS3-14	GAACATCATTACCATTATAA	C16G17	T16A17

Table S3B. Off target sites of TS3. Indels are shown in red capital letters. Whereas, PAM sites are shown in blue capital letters.

Line	Target sequence	Deletion	Insertion	Position
OT3-1	ACCATCGTAAAGG			
OS3-1	ACCATCTAAAGG	G7		LG7: 11322818-11322830
OS3-2	ACCATGGTAAAGG	C6	G6	LG7: 11322818-11322830
OS3-3	ACCATATTAAAGG	C6G7	A6T7	LG7: 11322818-11322830
OS3-4	ACCAATATAAAGG	T5C6G7	A5T6A7	LG7: 11322818-11322830
OS3-5	ACCATTTTAAAGG	C6G7	T6T7	LG7: 11322818-11322830
OS3-6	ACCGGAGTAAAGG	A4T5C6	G4G5A6	LG7: 11322818-11322830
OS3-7	ACCATCCTAAAGG	G7	C7	LG7: 11322818-11322830
OS3-8	ACCAATTAAAGG	T5C6G7	A5T6	LG7: 11322818-11322830
OS3-9	ACCATTGTAAAGG	C6	T6	LG11: 1787485-1787473
OS3-10	ACCGTAAAGG	A4T5C6		LG11: 1787485-1787473
OS3-11	ACCAAAGTAAAGG	T5C6	A5A6	LG11: 1787485-1787473
OS3-12	ACCATTATAAAGG	C6G7	T6A7	LG11: 1787485-1787473
OS3-13	ACCATAGTAAAGG	C6	A6	LG11: 1787485-1787473
OS3-14	ACCATCGTAAAGG	G7		LG11: 1787485-1787473
OS3-15	ACCCGAGTAAAGG	A4T5C6	C4G5A6	LG11: 1787485-1787473
OS3-16	ACCATAAAGG	T5C6G7		LG11: 1787485-1787473
OS3-17	ACCATGGTAAAGG	C6	G6	LG11: 1787485-1787473
OS3-18	ACCGAAGTAAAGG	A4T5C6G7	G4A5A6	LG15: 6006347-6006335
OS3-19	ACCAGAGTAAAGG	T5C6	G5A6	LG15: 6006347-6006335
OS3-20	ACCATCTTAAAGG	G7	T7	LG15: 6006347-6006335
OS3-21	ACCATTAAAGG	C6G7		LG15: 6006347-6006335

OS3-22	ACCAATAAAGG	T5C6G7	A5	LG15: 6006347-6006335
OS3-23	ACCAAAGTAAAGG	T5C6	A5A6	LG15: 6006347-6006335
OT3-2	ACCATCGTAACGG			
OS3-24	ACCATAGTAACGG	C6	A6	LG10: 335235-335223
OS3-25	ACCATCCTAACGG	G7	C7	LG10: 335235-335223
OS3-26	ACCAACTTAACGG	T5C6G7	A5C6T7	LG10: 335235-335223
OS3-27	ACCATAATAACGG	C6G7	A6A7	LG10: 335235-335223
OS3-28	ACCGCGGTAACGG	A4T5C6	G4C5G6	LG10: 335235-335223
OS3-29	ACCATCTAACGG	G7		LG10: 335235-335223
OT3-3	ACCATCGTAATGG			
OS3-30	ACCATTATAATGG	C6G7	T6A7	LG13: 4553200-4553212
OS3-31	ACCATTGTAATGG	C6	T6	LG13: 4553200-4553212
OS3-32	ACCAGATTAATGG	T5C6G7	G5A6T7	LG13: 4553200-4553212
OS3-33	ACCATCATAATGG	G7	A7	LG13: 4553200-4553212
OS3-34	ACCAAATTAATGG	T5C6G7	A5A6T7	LG13: 4553200-4553212
OS3-35	ACCATAGTAATGG	C6	A6	LG13: 4553200-4553212
OS3-36	ACCATTGTAATGG	C6	T6	LG13: 4553200-4553212

Table S4A. Target sequence of TS4 mutant lines. Indels are shown in red capital letters.

Line	Target sequence	Deletion	Insertion
Control	GCAATTTGTAACGTGATGGT		
TS4-1	GCAATTTGTAACGTGTGGT	A16	
TS4-2	GCAATTTGTAACGTGGT	T14G15A16	
TS4-3	GCAATTTGTAACGTGCGGT	A16T17	C16
TS4-4	GCAATTTGTAACGTGAAGGT	T17	A17
TS4-5	GCAATTTGTAACGTGCTGGT	A16	C16
TS4-6	GCAATTTGTAACGGGT	T14G15A16T17	
TS4-7	GCAATTTGTAACGCTGGT	T14G15A16	C14
TS4-8	GCAATTTGTAACGTGGGT	A16T17	
TS4-9	GCAATTTGTAACGTGAGGT	T17	
TS4-10	GCAATTTGTAACTGGT	G13T14G15A16	
TS4-11	GCAATTTGTAACGTGTTGGT	A16	T16
TS4-12	GCAATTTGTAACGGT	G13T14G15A16T17	
TS4-13	GCAATTTGTAACGCGGT	T14G15A16T17	C14
TS4-14	GCAATTTGTAACGTGGGGT	A16T17	G16
TS4-15	GCAATTTGTAACGCGTGGT	T14G15A16	C14G15
TS4-16	GCAATTTGTAACGTGACGGT	T17	C17
TS4-17	GCAATTTGTAACCCAGGT	G13T14G15A16T17	C13C14A15
TS4-18	GCAATTTGTAACGTGGTGGT	A16	G16
TS4-19	GCAATTTGTAACACCGGT	G13T14G15A16T17	A13C14C15
TS4-20	GCAATTTGTAACGCCGGGT	T14G15A16T17	C14C15G16
TS4-21	GCAATTTGTAACGTGGGGGT	A16T17	G16G17
TS4-22	GCAATTTGTAACGTGAGAGGT	T17	G17A18

TS4-23	GCAATTTGTAACCTGGT	G13T14G15A16	C13
TS4-24	GCAATTTGTAACGTGCGGGT	A16T17	C16G17
TS4-25	GCAATTTGTAACCGTGGT	G13T14G15A16	C13G14
TS4-26	GCAATTTGTAACCTGGT	G13T14G15A16	C13

Table S4B. Off target sites of TS4. Indels are shown in red capital letters. Whereas, PAM sites are shown in blue capital letters.

**Line**	**Target sequence**	**Deletion**	**Insertion**	**Position**
OT4-1	ACGTGATGGTAGG			
OS4-1	ACGTGAAGGTAGG	T7	A7	LG3: 35759-35747
OS4-2	ACGTGCGGGTAGG	A6T7	C6G7	LG3: 35759-35747
OS4-3	ACGTGTGGTAGG	A6		LG12: 3165551-3165539
OS4-4	ACGTCGGGGTAGG	G5A6T7	C5G6G7	LG12: 3165551-3165539
OS4-5	ACGTTGGTAGG	G5A6		LG12: 3165551-3165539
OS4-6	ACGTGACGGTAGG	T7	C7	LG13: 8362293-8362281
OS4-7	ACGTGGTAGG	G5A6T7		LG13: 8362293-8362281
OS4-8	ACGTGGGTAGG	A6T7		LG13: 8362293-8362281
OT4-2	ACGTGATGGTGGG			
OS4-9	ACGTGAAGGTGGG	T7	A7	LG13: 137165-137177
OS4-10	ACGCTTTGGTGGG	T4G5A6	C4T5T6	LG13: 137165-137177
OS4-11	ACGTCTTGGTGGG	G5A6	C5T6	LG23: 2807516-2807504
OS4-12	ACGTGATGGTGGG	T7		LG23: 2807516-2807504
OS4-13	ACGTCAGGGTGGG	G5A6T7	C5A6G7	LG23: 2807516-2807504
OT4-3	ACGTGATGGTCGG			
OS4-14	ACGTGCTGGTCGG	A6	C6	LG5: 13664324-13664312
OS4-15	ACGTGCCGGTCGG	A6T7	C6C7	LG5: 13664324-13664312
OT4-4	ACGTGATGGTTGG			
OS4-16	ACGTCCAGGTTGG	G5A6T7	C5C6A7	LG2: 2784141-2784129
OS4-17	ACGTGCGGGTTGG	A6T7	C6G7	LG2: 2784141-2784129
OS4-18	ACGTGAGGGTTGG	T7	G7	LG2: 2784141-2784129
OS4-19	ACGCAGTGGTTGG	T4G5A6	C4A5G6	LG2: 2784141-2784129
OS4-20	ACGTTCTGGTTGG	G5A6	T5C6	LG5: 1799204-1799216
OS4-21	ACGTGGTGGTTGG	A6	G6	LG5: 1799204-1799216
OS4-22	ACGATGTGGTTGG	T4G5A6	A4T5G6	LG5: 1799204-1799216
OS4-23	ACGTGGGGGTTGG	A6T7	G6G7	LG18: 711736-711724
OS4-24	ACGTGACGGTTGG	T7	C7	LG18: 711736-711724

Table S5A. Target sequence of TS5 mutant lines. Indels are shown in red capital letters.

**Line**	**Target sequence**	**Deletion**	**Insertion**
Control	GATCGATTATTGCGCTGTGG		
TS5-1	GATCGATTATTGCGCGTGG	T16	
TS5-2	GATCGATTATTGCGAGTGG	C15T16	A15
TS5-3	GATCGATTATTGCGCTCTGG	G17	C17

TS5-4	GATCGATTATTGCGGTGG	C15T16	
TS5-5	GATCGATTATTGCGC**CC**TGG	T16G17	C16C17
TS5-6	GATCGATTATTGCGC**A**GTGG	T16	A16
TS5-7	GATCGATTATTGCGTGG	G14C15T16	
TS5-8	GATCGATTATTGCGCTGG	T16G17	
TS5-9	GATCGATTATTGCGCT**A**TGG	G17	A17
TS5-10	GATCGATTATTGCTGG	G14C15T16G17	
TS5-11	GATCGATTATTGCG**GA**GTGG	C15T16	G15A16
TS5-12	GATCGATTATTGCGCT**T**TGG	G17	T17
TS5-13	GATCGATTATTGC**A**GTGG	G14C15T16	A14
TS5-14	GATCGATTATTGCGC**C**GTGG	T16	C16
TS5-15	GATCGATTATTGCGC**AC**TGG	T16G17	A16C17
TS5-16	GATCGATTATTGGTGG	C13G14C15T16	
TS5-17	GATCGATTATTGCG**AA**GTGG	C15T16	A15A16
TS5-18	GATCGATTATTGCGCT**AC**TGG	G17	A17C18
TS5-19	GATCGATTATTGC**AT**TGG	G14C15T16G17	A14T15
TS5-20	GATCGATTATTGCGC**AA**TGG	T16G17	A16A17
TS5-21	GATCGATTATTGC**AG**GTGG	G14C15T16	A14G15
TS5-22	GATCGATTATTGCGC**G**GTGG	T16	G16
TS5-23	GATCGATTATTGCGCTGTGG	T16	
TS5-24	GATCGATTATTGCGC**CA**TGG	T16G17	C16A17
TS5-25	GATCGATTATTGCGC**AA**TGG	T16G17	A16A17
TS5-26	GATCGATTATTGCGCTGTGG	T16	

Table S5B. Off target sites of TS5. Indels are shown in red capital letters. Whereas, PAM sites are shown in blue capital letters.

Line	Target sequence	Deletion	Insertion	Position
OT5-1	TGCGCTGTGG**CGG**			
OS5-1	TGCGCT**A**TGG**CGG**	G7	A7	LG3: 14717827-14717815
OS5-2	TGCGCTGG**CGG**	T6G7		LG3: 14717827-14717815
OS5-3	TGCGCGTGG**CGG**	T6		LG6: 5272386-5272398
OT5-2	TGCGCTGTGG**TGG**			
OS5-4	TGCG**AG**GTGG**TGG**	C5T6	A5G6	LG3: 306566-306554
OS5-5	TGCGCT**C**TGG**TGG**	G7	C7	LG3: 306566-306554
OS5-6	TGCG**A**TGG**TGG**	C5T6G7	A5	LG3: 306566-306554
OS5-7	TGCGC**AA**TGG**TGG**	T6G7	A6A7	LG6: 567561-567573
OS5-8	TGCGTGG**TGG**	G4C5T6		LG6: 567561-567573
OS5-9	TGCGC**C**GTGG**TGG**	T6	C6	LG6: 567561-567573
OS5-10	TGCG**AA**GTGG**TGG**	C5T6	A5A6	LG13: 821722-821710
OS5-11	TGCGTGG**TGG**	C5T6G7		LG13: 821722-821710
OS5-12	TGCGC**CA**TGG**TGG**	T6G7	C6A7	LG13: 821722-821710
OS5-13	TGCGCT**A**TGG**TGG**	G7	A7	LG17: 5383028-5383016

OT5-3	TGCGCTGTGG**AGG**			
OS5-14	TGCGC**A**GTGG**AGG**	T6	A6	LG9: 6175586-6175574
OS5-15	TGCGGTGG**AGG**	C5T6		LG9: 6175586-6175574
OS5-16	TGCG**ACC**TGG**AGG**	C5T6G7	A5C6C7	LG9: 6175586-6175574
OS5-17	TGCGCT**T**TGG**AGG**	G7	T7	LG9: 6175586-6175574
OS5-18	TGCGC**AC**TGG**AGG**	T6G7	A6C7	LG9: 6175586-6175574
OS5-19	TGC**AAG**GTGG**AGG**	G4C5T6	A4A5G6	LG11: 6175574-6345549
OS5-20	TGCGTGG**AGG**	C5T6G7		LG11: 6175574-6345549
OS5-21	TGCG**GA**GTGG**AGG**	C5T6	G5A6	LG11: 6175574-6345549
OS5-22	TGCGCTTGG**AGG**	G7		LG11: 6175574-6345549
OS5-23	TGCGC**C**GTGG**AGG**	T6	C6	LG11: 6175574-6345549
OS5-24	TGCGCTGG**AGG**	T6G7		LG14: 6340613-6340601

Table S6A. Target sequence of TS6 mutant lines. Indels are shown in red capital letters.

Line	Target sequence	Deletion	Insertion
Control	GTTATTGACGGTTAATGTGC		
TS6-1	GTTATTGACGGTTAA**A**TGC	T16G17	A16
TS6-2	GTTATTGACGGTTAAT**C**TGC	G17	C17
TS6-3	GTTATTGACGGTTAA**A**GTGC	T16	A16
TS6-4	GTTATTGACGGTTA**C**TGC	A15T16G17	C15
TS6-5	GTTATTGACGGTTAATGC	T16G17	
TS6-6	GTTATTGACGGTTAA**C**GTGC	T16	C16
TS6-7	GTTATTGACGGTT**G**GTGC	A14A15T16	G14
TS6-8	GTTATTGACGGTTATGC	A15T16G17	
TS6-9	GTTATTGACGGTTAATTGC	G17	
TS6-10	GTTATTGACGGTT**C**GTGC	A14A15T16	C14
TS6-11	GTTATTGACGGTTAA**C**TGC	T16G17	C16
TS6-12	GTTATTGACGGTTAA**G**GTGC	T16	G16
TS6-13	GTTATTGACGGTTA**CA**TGC	A15T16G17	C15A16
TS6-14	GTTATTGACGGTTAAT**A**TGC	G17	A17
TS6-15	GTTATTGACGGTT**CG**GTGC	A14A15T16	C14G15
TS6-16	GTTATTGACGGTTAAGTGC	T16	
TS6-17	GTTATTGACGGT**C**GTGC	T13A14A15T16	C13
TS6-18	GTTATTGACGGTTAA**AA**TGC	T16G17	A16A17
TS6-19	GTTATTGACGGTTAAT**T**TGC	G17	T17
TS6-20	GTTATTGACGGTTAATGTGC	T12T13A14A15	
TS6-21	GTTATTGACGGTGTGC	T13A14A15T16	
TS6-22	GTTATTGACGGTTAA**AC**GTGC	T16	A16C17
TS6-23	GTTATTGACGGT**GA**GTGC	T13A14A15T16	G13A14
TS6-24	GTTATTGACGGTTAA**CA**TGC	T16G17	C16A17
TS6-25	GTTATTGACGGTTAAT**AT**TGC	G17	A17T18
TS6-26	GTTATTGACGGTTA**CT**TGC	A15T16G17	C15T16
TS6-27	GTTATTGACGGTTAA**AC**TGC	T16G17	A16C17

Table S6B. Off target sites of TS6. Indels are shown in red capital letters. Whereas, PAM sites are shown in blue capital letters.

Line	Target sequence	Deletion	Insertion	Position
OT6-1	GTTAATGTGCTGG			
OS6-1	GTTAATTGCTGG	G7		LG7: 1729787-1729799
OS6-2	GTTAACATGCTGG	T6G7	C6A7	LG7: 1729787-1729799
OS6-3	GTTAAAGTGCTGG	T6	A6	LG7: 1729787-1729799
OS6-4	GTTACGGTGCTGG	A5T6	C5G6	LG7: 1729787-1729799
OS6-5	GTTAATATGCTGG	G7	A7	LG15: 1940260-1940248
OS6-6	GTTATGCTGG	A5T6G7		LG15: 1940260-1940248
OS6-7	GTTCCGGTGCTGG	A4A5T6	C4C5G6	LG15: 1940260-1940248
OS6-8	GTTAACGTGCTGG	T6	C6	LG15: 1940260-1940248
OS6-9	GTTAGTGCTGG	A5T6		LG15: 1940260-1940248
OS6-10	GTTAACCTGCTGG	T6G7	C6C7	LG24: 285191-285203
OS6-11	GTTCAGGTGCTGG	A4A5T6	C4A5G6	LG24: 285191-285203
OS6-12	GTTACCTGCTGG	A5T6G7	C5C6	LG24: 285191-285203
OS6-13	GTTAATCTGCTGG	G7	C7	LG24: 285191-285203
OS6-14	GTTGCGTGCTGG	A4A5T6	G4C5	LG24: 285191-285203
OS6-15	GTTAGCGTGCTGG	A5T6	G5C6	LG24: 285191-285203
OT6-2	GTTAATGTGCGGG			
OS6-16	GTTAAGGTGCGGG	T6	G6	LG5: 5800955-5800943
OS6-17	GTTACAATGCGGG	A5T6G7	C5A6A7	LG5: 5800955-5800943
OS6-18	GTTAATTTGCGGG	G7	T7	LG5: 5800955-5800943
OS6-19	GTTAATGCGGG	T6G7		LG5: 5800955-5800943
OT6-3	GTTAATGTGCAGG			
OS6-20	GTTATGCAGG	A5T6G7		LG1: 1429364-1429352
OS6-21	GTTACCGTGCAGG	A5T6	C5C6	LG1: 1429364-1429352
OS6-22	GTTAATTGCAGG	G7		LG1: 1429364-1429352
OS6-23	GTTAAAATGCAGG	T6G7	A6A7	LG5: 2942184-2942196

Table S7A. Target sequence of TS7 mutant lines. Indels are shown in red capital letters.

Line	Target sequence	Deletion	Insertion
Control	GTGGATCAAGAATCACCCGG		
TS7-1	GTGGATCAAGAATCACGCGG	C17	G17
TS7-2	GTGGATCAAGAATCACGG	C16C17	
TS7-3	GTGGATCAAGAATCACCGG	C16	
TS7-4	GTGGATCAAGAATCGCGG	A15C16C17	G15
TS7-5	GTGGATCAAGAATCACCGG	C17	
TS7-6	GTGGATCAAGAATTCCGG	C14A15C16	T14
TS7-7	GTGGATCAAGAATCAACGG	C16C17	A16
TS7-8	GTGGATCAAGAATCATCCGG	C16	T16
TS7-9	GTGGATCAAGAATTCGG	C14A15C16C17	T14
TS7-10	GTGGATCAAGAATCACACGG	C17	A17
TS7-11	GTGGATCAAGAATCCGG	C14A15C16	

TS7-12	GTGGATCAAGAATCAGCGG	C16C17	G16
TS7-13	GTGGATCAAGAACCGG	T13C14A15C16	
TS7-14	GTGGATCAAGAATCCGG	A15C16C17	
TS7-15	GTGGATCAAGAATCAACCGG	C16	A16
TS7-16	GTGGATCAAGAATCACATCGG	C17	A17T18
TS7-17	GTGGATCAAGAAGTCCGG	T13C14A15C16	G13T14
TS7-18	GTGGATCAAGAATCATCGG	C16C17	T16
TS7-19	GTGGATCAAGAATCAGCCGG	C16	G16
TS7-20	GTGGATCAAGAATGGCCGG	C14A15C16	G14G15
TS7-21	GTGGATCAAGAATCACTCGG	C17	T17
TS7-22	GTGGATCAAGAATCGG	C14A15C16C17	
TS7-23	GTGGATCAAGAATCTCGG	A15C16C17	T15
TS7-24	GTGGATCAAGAATCAAGCGG	C16C17	A16G17
TS7-25	GTGGATCAAGAATCATTCCGG	C16	T16T17
TS7-26	GTGGATCAAGAATCACGACGG	C17	G17A18
TS7-27	GTGGATCAAGAAGCCGG	T13C14A15C16	G13
TS7-28	GTGGATCAAGAATCGTCGG	A15C16C17	G15T16
TS7-29	GTGGATCAAGAGAACCGG	A12T13C14A15C16	G12A13A14
TS7-30	GTGGATCAAGAATCAGGCGG	C16C17	G16G17
TS7-31	GTGGATCAAGAATCAGACCGG	C16	G16A17
TS7-32	GTGGATCAAGAGGCCGG	A12T13C14A15C16	G12G13
TS7-33	GTGGATCAAGAATTGCCGG	C14A15C16	T14G15
TS7-34	GTGGATCAAGAATCACTGCGG	C17	T17G18

Table S7B. Off target sites of TS7. Indels are shown in red capital letters. Whereas, PAM sites are shown in blue capital letters.

Line	Target sequence	Deletion	Insertion	Position
OT7-1	AATCACCCGGAGG			
OS7-1	AATCACACGGAGG	C7	A7	LG4: 15026622-15026610
OS7-2	AATCATCCGGAGG	C6	T6	LG4: 15026622-15026610
OS7-3	AATCAAGCGGAGG	C6C7	A6G7	LG4: 15026622-15026610
OS7-4	AATCCGGAGG	A5C6C7		LG4: 15026622-15026610
OS7-5	AATCACCGGAGG	C6		LG4: 15026622-15026610
OS7-6	AATCACGCGGAGG	C7	G7	LG21: 9932008-9932020
OS7-7	AATCGAGCGGAGG	A5C6C7	G5A6G7	LG21: 9932008-9932020
OS7-8	AATCAGTCGGAGG	C6C7	G6T7	LG21: 9932008-9932020
OS7-9	AATTTGCCGGAGG	C4A5C6	T4T5G6	LG21: 9932008-9932020
OT7-2	AATCACCCGGGGG			
OS7-10	AATCAGCCGGGGG	C6	G6	LG17: 9777375-9777387
OS7-11	AATCACTCGGGGG	C7	T7	LG17: 9777375-9777387
OS7-12	AATCTGACGGGGG	A5C6C7	T5G6A7	LG17: 9777375-9777387
OS7-13	AATCAATCGGGGG	C6C7	A6T7	LG17: 9777375-9777387
OS7-14	AATCACGCGGGGG	C7	G7	LG17: 9777375-9777387

OS7-15	AATCA**GT**CGG**GGG**	C6C7	G6T7	LG17: 9777375-9777387
OS7-16	AATCA**A**CCGG**GGG**	C6	A6	LG17: 9777375-9777387

Table S8A. Target sequence of TS8 mutant lines. Indels are shown in red capital letters.

Line	Target sequence	Deletion	Insertion
Control	TCAAAGGACTTCGGCCTCCC		
TS8-1	TCAAAGGACTTCGGC**A**TCCC	C16	A16
TS8-2	TCAAAGGACTTCGGCCCC	C16T17	
TS8-3	TCAAAGGACTTCGGCC**A**CCC	T17	A17
TS8-4	TCAAAGGACTTCGGTCCC	C15C16	
TS8-5	TCAAAGGACTTCG**AGG**TCCC	G14C15C16	A14G15G16
TS8-6	TCAAAGGACTTCGGC**AC**CCC	C16T17	A16C17
TS8-7	TCAAAGGACTTCGGC**G**TCCC	C16	G16
TS8-8	TCAAAGGACTTC**A**CCC	G13G14C15C16T17	A13
TS8-9	TCAAAGGACTTCGGCCCCC	T17	
TS8-10	TCAAAGGACTTC**AG**CTCCC	G13G14C15	A13G14
TS8-11	TCAAAGGACTTCGGC**GA**CCC	C16T17	G16A17
TS8-12	TCAAAGGACTTCG**AG**TCCC	G14C15C16	A14G15
TS8-13	TCAAAGGACTTCGGCC**G**CCC	T17	G17
TS8-14	TCAAAGGACTTCGG**AT**TCCC	C15C16	A15T16
TS8-15	TCAAAGGACTTCGGCTCCC	C16	
TS8-16	TCAAAGGACTTCCCC	G13G14C15C16T17	
TS8-17	TCAAAGGACTTCGGC**G**CCC	C16T17	G16
TS8-18	TCAAAGGACTTCGGCC**C**CCC	T17	C17
TS8-19	TCAAAGGACTT**AT**TCCC	C12G13G14C15C16	A12T13
TS8-20	TCAAAGGACTTCTCCC	G13G14C15C16	
TS8-21	TCAAAGGACTTCGGC**T**TCCC	C16	T16
TS8-22	TCAAAGGACTT**TTAAG**TCCC	C12G13G14C15C16	T12T13A14A15G16
TS8-23	TCAAAGGACTTCGGCC**AG**CCC	T17	A17G18
TS8-24	TCAAAGGACTTCGG**G**TCCC	C15C16	G15
TS8-25	TCAAAGGACTTCGGC**AG**CCC	C16T17	A16G17
TS8-26	TCAAAGGACTTC**T**CTCCC	G13G14C15	T13
TS8-27	TCAAAGGACTTCG**TAG**TCCC	G14C15C16	T14A15G16
TS8-28	TCAAAGGACTTCGG**TG**TCCC	C15C16	T15G16
TS8-29	TCAAAGGACTTCGGC**AG**TCCC	C16	A16G17
TS8-30	TCAAAGGACTTC**ATAT**TCCC	G13G14C15C16	A13T14A15T16
TS8-31	TCAAAGGACTTCGG**AT**TCCC	C15C16	A15T16
TS8-32	TCAAAGGACTTCGGC**AA**CCC	C16T17	A16A17
TS8-33	TCAAAGGACTTC**AGA**CCC	G13G14C15C16T17	A13G14A15
TS8-34	TCAAAGGACTTCGGCC**GC**CCC	T17	G17C18
TS8-35	TCAAAGGACTTCGGC**GG**TCCC	C16	G16G17
TS8-36	TCAAAGGACTT**ATAAT**TCCC	C12G13G14C15C16	A12T13A14A15T16

Table S8B. Off target sites of TS8. Indels are shown in red capital letters. Whereas, PAM sites are shown in blue capital letters.

Line	Target sequence	Deletion	Insertion	Position
OT8-1	TCGGCCTCCC**CGG**			
OS8-1	TCGGCCCCC**CGG**	T7		LG4: 15099752-15099740
OS8-2	TCGGC**A**TCCC**CGG**	C6	A6	LG4: 15099752-15099740
OS8-3	TCGGCCCC**CGG**	C6T7		LG4: 15099752-15099740
OS8-4	TCGG**GA**TCCC**CGG**	C5C6	G5A6	LG4: 15099752-15099740
OS8-5	TCGGCC**G**CCC**CGG**	T7	G7	LG9: 1320081-1320093
OS8-6	TCGG**AG**CCC**CGG**	C5C6T7	A5G6	LG9: 1320081-1320093
OS8-7	TCGGC**G**TCCC**CGG**	C6	G6	LG9: 1320081-1320093
OS8-8	TCGGC**AG**CCC**CGG**	C6T7	A6G7	LG9: 1320081-1320093
OS8-9	TCGG**GC**CCC**CGG**	C5C6T7	G5C6	LG9: 1320081-1320093
OT8-2	TCGGCCTCCC**GGG**			
OS8-10	TCGGCC**A**CCC**GGG**	T7	A7	LG3: 3864117-3864105
OS8-11	TCGGC**T**TCCC**GGG**	C6	T6	LG3: 3864117-3864105
OS8-12	TCGG**AA**TCCC**GGG**	C5C6	A5A6	LG3: 3864117-3864105
OS8-13	TCGGCC**C**CCC**GGG**	T7	C7	LG3: 3864117-3864105
OS8-14	TCGGC**AG**CCC**GGG**	C6T7	A6G7	LG3: 3864117-3864105

Table S9A. Target sequence of TS9 mutant lines. Indels are shown in red capital letters.

Line	Target sequence	Deletion	Insertion
Control	AGAGGCTGTCGGAGAGGCAC		
TS9-1	AGAGGCTGTCGGAGAGCAC	G16	
TS9-2	AGAGGCTGTCGGAGAAGCAC	G16	A16
TS9-3	AGAGGCTGTCGGAGAGCCAC	G17	C17
TS9-4	AGAGGCTGTCGGAGAGCAC	G17	
TS9-5	AGAGGCTGTCGGAGAGTCAC	G17	T17
TS9-6	AGAGGCTGTCGGAGACAC	G16G17	
TS9-7	AGAGGCTGTCGGAGACCAC	G16G17	C16
TS9-8	AGAGGCTGTCGGAGGGCAC	A15	
TS9-9	AGAGGCTGTCGGAGTGGCAC	A15	T15
TS9-10	AGAGGCTGTCGGAAGGCAC	G14	
TS9-11	AGAGGCTGTCGGACAGGCAC	G14	C14
TS9-12	AGAGGCTGTCGGAAAGGCAC	G14	A14
TS9-13	AGAGGCTGTCGGAGCAC	G14A15G16	
TS9-14	AGAGGCTGTCGGATGCAC	G14A15G16	T14
TS9-15	AGAGGCTGTCGGAGCAC	A15G16G17	
TS9-16	AGAGGCTGTCGGAGCCAC	A15G16G17	C15
TS9-17	AGAGGCTGTCGGAGTCAC	A15G16G17	T15
TS9-18	AGAGGCTGTCGGAGGCAC	A15G16	
TS9-19	AGAGGCTGTCGGAGCGCAC	A15G16	C15
TS9-20	AGAGGCTGTCGGAGTGCAC	A15G16	T15
TS9-21	AGAGGCTGTCGGAGCCGCAC	A15G16	C15C16
TS9-22	AGAGGCTGTCGGAGAGCCCAC	G16	C16C17
TS9-23	AGAGGCTGTCGGAGACGCAC	G16	C16
TS9-24	AGAGGCTGTCGGAGATGCAC	G16	T16
TS9-25	AGAGGCTGTCGGAGATCAC	G16G17	T16
TS9-26	AGAGGCTGTCGGAGAACAC	G16G17	A16
TS9-27	AGAGGCTGTCGGAGACACAC	G16G17	C16A17
TS9-28	AGAGGCTGTCGGAGAGACAC	G17	A17
TS9-29	AGAGGCTGTCGGAGAGCCCAC	G17	C17C18
TS9-30	AGAGGCTGTCGGAGACTCAC	G16G17	C16T17
TS9-31	AGAGGCTGTCGGAGATACAC	G16G17	T16A17
TS9-32	AGAGGCTGTCGGACGCAC	G14A15G16	C14
TS9-33	AGAGGCTGTCGGACCGCAC	G14A15G16	C14C15

Table S9B. Off target sites of TS9. Indels are shown in red capital letters. Whereas, PAM sites are shown in blue capital letters.

Line	Target sequence	Deletion	Insertion	Position
OT9-1	GGAGAGGCACAGG			
OS9-1	GGAGAGACACAGG	G7	A7	LG3: 2582124-2582136
OS9-2	GGAGATGCACAGG	G6	T6	LG3: 2582124-2582136

OS9-3	GGAGACAC**AGG**	G6G7		LG3: 2582124-2582136
OS9-4	GGAGAG**C**CAC**AGG**	G7	C7	LG3: 2582124-2582136
OS9-5	GGAG**CT**GCAC**AGG**	A5G6	C5T6	LG4: 363763-363775
OS9-6	GGA**CC**GCAC**AGG**	G4A5G6	C4C5	LG4: 363763-363775
OT9-2	GGAG**AGG**CAC**CGG**			
OS9-7	GGAGAG**C**CAC**CGG**	G6	C6	LG5: 64475-64487
OS9-8	GGAGAG**A**CAC**CGG**	G7	A7	LG5: 64475-64487
OS9-9	GGAG**TT**GCAC**CGG**	A5G6	T5T6	LG5: 64475-64487
OS9-10	GGAGA**AC**CAC**CGG**	G6G7	A6C7	LG20: 3568834-3568846
OS9-11	GGA**TTC**GCAC**CGG**	G4A5G6	T4T5C6	LG20: 3568834-3568846
OT9-3	GGAG**AGG**CAC**GGG**			
OS9-12	GGAGAG**T**CAC**GGG**	G7	T7	LG1: 11859426-11859414
OS9-13	GGAGAG**A**CAC**GGG**	G7	A7	LG1: 11859426-11859414
OS9-14	GGAG**T**GCAC**GGG**	A5G6	T5	LG24: 1923013-1923025
OS9-15	GGAGAG**A**CAC**GGG**	G6	A6	LG24: 1923013-1923025
OS9-16	GGAGA**CT**CAC**GGG**	G6G7	C6T7	LG24: 1923013-1923025
OS9-17	GGAGA**CA**CAC**GGG**	G6G7	C6A7	LG24: 1923013-1923025

Table S10A. Target sequence of TS10 mutant lines. Indels are shown in red capital letters.

**Line**	**Target sequence**	**Deletion**	**Insertion**
Control	ATCGCCACCAACGGCCGCCA		
TS10-1	ATCGCCACCAACGGCGCCA	C16	
TS10-2	ATCGCCACCAACGGCC**A**CCA	G17	A17
TS10-3	ATCGCCACCAACGGCC**T**CCA	G17	T17
TS10-4	ATCGCCACCAACGGC**T**GCCA	C16	T16
TS10-5	ATCGCCACCAACGGCCCA	C16G17	
TA10-6	ATCGCCACCAACGGCC**C**CCA	G17	C17
TS10-7	ATCGCCACCAACGGC**A**CCA	C16G17	A16
TS10-8	ATCGCCACCAACGGC**A**GCCA	C16	A16
TS10-9	ATCGCCACCAACGGC**T**CCA	C16G17	T16
TS10-10	ATCGCCACCAACGGC**G**GCCA	C16	G16
TS10-11	ATCGCCACCAACGGCC**AA**CCA	G17	A17A18
TS10-12	ATCGCCACCAACGGC**TA**CCA	C16G17	T16A17
TS10-13	ATCGCCACCAACGGCC**AT**CCA	G17	A17T18
TS10-14	ATCGCCACCAACGGC**AA**GCCA	C16	A16A17
TS10-15	ATCGCCACCAACGGGCCA	C15C16	
TS10-16	ATCGCCACCAACGGC**TG**GCCA	C16	T16G17
TS10-17	ATCGCCACCAACGG**T**GCCA	C15C16	T15
TS10-18	ATCGCCACCAACGGCC**CC**CCA	G17	C17C18
TS10-19	ATCGCCACCAACGG**A**GCCA	C15C16	A15
TS10-20	ATCGCCACCAACGG**T**CCA	C15C16G17	T15
TS10-21	ATCGCCACCAACGCGCCA	G14C15	
TS10-22	ATCGCCACCAACGGC**TT**GCCA	C16	T16T17

TS10-23	ATCGCCACCAACGGCCACCCA	G17	A17C18
TS10-24	ATCGCCACCAACGGTCCA	C15C16G17	T15
TS10-25	ATCGCCACCAACGGCAAGCCA	C16	A16A17
TS10-26	ATCGCCACCAACGGTGCCA	C15C16	T15

Table S10B. Off target sites of TS10. Indels are shown in red capital letters. Whereas, PAM sites are shown in blue capital letters.

**Line**	**Target sequence**	**Deletion**	**Insertion**	**Position**
OT10-1	ACGGCCGCCA**GGG**			
OS10-1	ACGGCCCCA**GGG**	G7		LG4: 4331720-4331708
OS10-2	ACGGC**AA**CCA**GGG**	C6G7	A6A7	LG4: 4331720-4331708
OS10-3	ACGGCGCCA**GGG**	C6		LG4: 4331720-4331708
OS10-4	ACGGCC**A**CCA**GGG**	G7	A7	LG4: 4331720-4331708
OS10-5	ACGGGCCA**GGG**	C5C6		LG4: 4331720-4331708
OS10-6	ACG**AAT**GCCA**GGG**	G4C5C6	A4A5T6	LG9: 1320900-1320912
OS10-7	ACGGC**A**GCCA**GGG**	C6	A6	LG9: 1320900-1320912
OS10-8	ACGGC**TA**CCA**GGG**	C6G7	T6A7	LG9: 1320900-1320912
OS10-9	ACGGCC**T**CCA**GGG**	G7	T7	LG9: 1320900-1320912
OT10-2	ACGGCCGCCA**AGG**			
OS10-10	ACGG**AT**GCCA**AGG**	C5C6	A5T6	LG4: 10316230-10316218
OS10-11	ACGGC**GA**CCA**AGG**	C6G7	G6A7	LG4: 10316230-10316218
OS10-12	ACGGC**T**GCCA**AGG**	C6	T6	LG4: 10316230-10316218
OS10-13	ACG**ATT**GCCA**AGG**	G4C5C6	A4T5T6	LG4: 10316230-10316218
OT10-3	ACGGCCGCCA**TGG**			
OS10-14	ACGGCC**C**CCA**TGG**	G7	C7	LG16: 8923099-8923087
OS10-15	ACGG**TT**GCCA**TGG**	C5C6	T5T6	LG16: 8923099-8923087
OS10-16	ACGGCCCA**TGG**	C6G7		LG16: 8923099-8923087
OS10-17	ACGGC**G**GCCA**TGG**	C6	G6	LG16: 8923099-8923087
OS10-18	ACG**TTG**GCCA**TGG**	G4C5C6G7	T4T5G6	LG18: 9372049-9372061
OS10-19	ACGGCC**A**CCA**TGG**	G7	A7	LG18: 9372049-9372061
OS10-20	ACG**CTA**GCCA**TGG**	G4C5C6	C4T5A6	LG25: 3379815-3379827
OS10-21	ACGGC**T**GCCA**TGG**	C6	T6	LG25: 3379815-3379827
OT10-4	ACGGCCGCCA**CGG**			
OS10-22	ACGGCGCCA**CGG**	C6		LG20: 5695523-5695511
OS10-23	ACGG**GA**GCCA**CGG**	C5C6	G5A6	LG20: 5695523-5695511
OS10-24	ACGGC**TT**CCA**CGG**	C6G7	T6T7	LG20: 5695523-5695511

Table S11A. Target sequence of TS11 mutant lines. Indels are shown in red capital letters.

**Line**	**Target sequence**	**Deletion**	**Insertion**
Control	GCGCGAAGAGGCTGTCGGAG		
TS11-1	GCGCGAAGAGGCTGTCGAG	G17	
TS11-2	GCGCGAAGAGGCTGTCAGAG	G17	A17
TS11-3	GCGCGAAGAGGCTGTGGGAG	C16	G16

TS11-4	GCGCGAAGAGGCTGTTGAG	C16G17	T16
TS11-5	GCGCGAAGAGGCTGGGAG	T15C16	
TS11-6	GCGCGAAGAGGCTGTCTGAG	G17	T17
TS11-7	GCGCGAAGAGGCTGTGGAG	C16	
TS11-8	GCGCGAAGAGGCTGAGGAG	T15C16	A15
TS11-9	GCGCGAAGAGGCTGTCCGAG	G17	C17
TS11-10	GCGCGAAGAGGCTGTGAG	C16G17	
TS11-11	GCGCGAAGAGGCTGGAG	G14T15C16	
TS11-12	GCGCGAAGAGGCTGTCATGAG	G17	A17T18
TS11-13	GCGCGAAGAGGCTAGGAG	G14T15C16	A14
TS11-14	GCGCGAAGAGGCTGTAGGAG	C16	A16
TS11-15	GCGCGAAGAGGCTGTTAGAG	C16G17	T16A17
TS11-16	GCGCGAAGAGGCTAGGGAG	G14T15C16	A14G15
TS11-17	GCGCGAAGAGGCTGGGGAG	T15C16	G15
TS11-18	GCGCGAAGAGGCTGTCAAGAG	G17	A17A18
TS11-19	GCGCGAAGAGGCTTAGGAG	G14T15C16	T14A15
TS11-20	GCGCGAAGAGGCTGTAAGGAG	C16	A16A17
TS11-21	GCGCGAAGAGGCTGTCCAGAG	G17	C17A18
TS11-22	GCGCGAAGAGGCTGTATGAG	C16G17	A16T17
TS11-23	GCGCGAAGAGGCTAAGGAG	G14T15C16	A14A15
TS11-24	GCGCGAAGAGGCTGTTCGGAG	C16	T16C17
TS11-25	GCGCGAAGAGGCTTAGGAG	G14T15C16	T14A15

Table S11B. Off target sites of TS11. Indels are shown in red capital letters. Whereas, PAM sites are shown in blue capital letters

Line	Target sequence	Deletion	Insertion	Position
OT11-1	GCTGTCGGAGAGG			
OS11-1	GCTGTCTGAGAGG	G7	T7	LG12: 3408085-3408097
OS11-2	GCTGTAGGAGAGG	C6	A6	LG12: 3408085-3408097
OS11-3	GCTGTGAGAGG	C6G7		LG12: 3408085-3408097
OS11-4	GCTGAGGGAGAGG	T5C6	A5G6	LG12: 3408085-3408097
OS11-5	GCTGTCAGAGAGG	G7	A7	LG16: 3427010-3427022
OS11-6	GCTGGAGAGG	T5C6G7		LG16: 3427010-3427022
OS11-7	GCTGTATGAGAGG	C6G7	A6T7	LG16: 3427010-3427022
OS11-8	GCTGTTGGAGAGG	C6	T6	LG20: 4912655-4912643
OS11-9	GCTGTCCGAGAGG	G7	C7	LG20: 4912655-4912643
OT11-2	GCTGTCGGAGGGG			
OS11-10	GCTGGAGGAGGGG	T5C6	G5A6	LG2: 2037308-2037296
OS11-11	GCTGTGGGAGGGG	C6	G6	LG2: 2037308-2037296
OS11-12	GCTGTCAGAGGGG	G7	A7	LG2: 2037308-2037296
OS11-13	GCTGATGAGGGG	T5C6G7	A5T6	LG25: 3396163-3396151
OT11-3	GCTGTCGGAGCGG			
OS11-14	GCTGTTTGAGCGG	C6G7	T6T7	LG1: 227015-227027
OS11-15	GCTGTTGGAGCGG	C6	T6	LG1: 227015-227027

OS11-16	GCTGTCTGAGCGG	G7	T7	LG1: 227015-227027
OS11-17	GCTGAAGAGCGG	T5C6G7	A5A6	LG12: 2887376-2887388
OS11-18	GCTGGCGAGCGG	T5C6G7	G5C6	LG12: 2887376-2887388
OS11-19	GCTGGGGGAGCGG	T5C6	G5G6	LG12: 2887376-2887388
OS11-20	GCTGTATGAGCGG	C6G7	A6T7	LG12: 2887376-2887388
OS11-21	GCTGTGGAGCGG	C6		LG19: 7144935-7144947
OT11-4	GCTGTCGGAGTGG			
OS11-22	GCTGTAGGAGTGG	C6	A6	LG3: 2110153-2110165
OS11-23	GCTGCAGAGTGG	T5C6G7	C5A6	LG3: 2110153-2110165
OS11-24	GCTGTAAGAGTGG	C6G7	A6A7	LG10: 8457549-8457537
OS11-25	GCTGTCGAGTGG	G7		LG23: 1526853-1526865

Table S12A. Target sequence of TS12 mutant lines. Indels are shown in red capital letters.

**Line**	**Target sequence**	**Deletion**	**Insertion**
Control	ATTATAGGTTACTTGTACCC		
TS12-1	ATTATAGGTTACTTGTCCC	A17	
TS12-2	ATTATAGGTTACTTGAACCC	T16	A16
TS12-3	ATTATAGGTTACTTACCC	G15T16	
TS12-4	ATTATAGGTTACTTGCCCC	T16A17	C16
TS12-5	ATTATAGGTTACTTAACCC	G15T16	A15
TS12-6	ATTATAGGTTACTTGTTCCC	A17	T17
TS12-7	ATTATAGGTTACTACCC	T14G15T16	
TS12-8	ATTATAGGTTACTTGCACCC	T16	C16
TS12-9	ATTATAGGTTACTAACCC	T14G15T16	A14
TS12-10	ATTATAGGTTACTTGCCC	T16A17	
TS12-11	ATTATAGGTTACTTCACCC	G15T16	C15
TS12-12	ATTATAGGTTACTTGTGCCC	A17	G17
TS12-13	ATTATAGGTTACTTACCC	T14G15	
TS12-14	ATTATAGGTTACTTGGCCC	T16A17	G16
TS12-15	ATTATAGGTTACTTGACCC	T16	
TS12-16	ATTATAGGTTACTATACCC	T14G15	A14
TS12-17	ATTATAGGTTACTTGTCCCC	A17	C17
TS12-18	ATTATAGGTTACTCACCC	T14G15T16	C14
TS12-19	ATTATAGGTTACTTAAACCC	G15T16	A15A16
TS12-20	ATTATAGGTTACTTGCGCCC	T16A17	C16G17
TS12-21	ATTATAGGTTACTTGACACCC	T16	A16C17
TS12-22	ATTATAGGTTACTACACCC	T14G15T16	A14C15
TS12-23	ATTATAGGTTACACCC	T13T14G15T16	
TS12-24	ATTATAGGTTACTTGCCCCC	T16A17	C16C17
TS12-25	ATTATAGGTTACTTGGCACCC	T16	G16C17
TS12-26	ATTATAGGTTACAACCC	T13T14G15T16	A13

Table S12B. Off target sites of TS12. Indels are shown in red capital letters. Whereas, PAM

sites are shown in blue capital letters.

Line	Target sequence	Deletion	Insertion	Position
OT12-1	ACTTGTACCCTGG			
OS12-1	ACTTGTTCCCTGG	A7	T7	LG7: 9743362-9743350
OS12-2	ACTTACCCTGG	G5T6		LG7: 9743362-9743350
OS12-3	ACTTGCTCCCTGG	T6A7	C6T7	LG7: 9743362-9743350
OS12-4	ACTTGAACCCTGG	T6	A6	LG7: 9743362-9743350
OS12-5	ACTTCCCTGG	G5T6A7		LG7: 9743362-9743350
OS12-6	ACTTGTGCCCTGG	A7	G7	LG11: 7905958-7905946
OS12-7	ACTTCCACCCTGG	G5T6	C5C6	LG11: 7905958-7905946
OS12-8	ACTTCTCCCTGG	G5T6A7	C5T6	LG11: 7905958-7905946
OS12-9	ACTTGCCCCCTGG	T6A7	C6C7	LG11: 7905958-7905946
OS12-10	ACTTCAACCCTGG	G5T6	C5A6	LG11: 7905958-7905946
OS12-11	ACTTGCACCCTGG	T6	C6	LG13: 9285076-9285064
OS12-12	ACTTCCGCCCTGG	G5T6A7	C5C6G7	LG13: 9285076-9285064
OS12-13	ACTTGTCCCCTGG	A7	C7	LG13: 9285076-9285064
OS12-14	ACTTACACCCTGG	G5T6	A5C6	LG13: 9285076-9285064
OS12-15	ACTTGGCCCCTGG	T6A7	G6C7	LG13: 9285076-9285064
OT12-2	ACTTGTACCCCGG			
OS12-16	ACTTGTTCCCCGG	A7	T7	LG15: 5553406-5553394
OS12-17	ACTTGCGCCCCGG	T6A7	C6G7	LG15: 5553406-5553394
OS12-18	ACTTGGACCCCGG	T6	G6	LG15: 5553406-5553394
OS12-19	ACTTACCCCCGG	G5T6A7	A5C6	LG15: 5553406-5553394
OS12-20	ACTTGTCCCCGG	A7		LG15: 5553406-5553394
OS12-21	ACTTAAACCCCGG	G5T6	A5A6	LG15: 5591597-5591585
OS12-22	ACTTACACCCCGG	G5T6	A5C6	LG15: 5591597-5591585
OS12-23	ACTTGGGCCCCGG	T6A7	G6G7	LG15: 5591597-5591585
OS12-24	ACTTGGACCCCGG	T6	G6	LG15: 5591597-5591585

Table S13A. Target sequence of TS13 mutant lines. Indels are shown in red capital letters.

Line	Target sequence	Deletion	DA	Insertion	IA
Control	GTCAAGCTCAATGTGTCCCC				
TS13-1	GTCAAGCTCAATGTGTACCC	C17		A17	
TS13-2	GTCAAGCTCAATGTGCCCCC	T16		C16	
TS13-3	GTCAAGCTCAATGTGCCC	T16C17			
TS13-4	GTCAAGCTCAATGTGTCCC	C17			
TS13-5	GTCAAGCTCAATGTACCCC	G15T16		A15	
TS13-6	GTCAAGCTCAATGTGCCCC	T16			
TS13-7	GTCAAGCTCAATGTGACCC	T16C17		A16	
TS13-8	GTCAAGCTCAATGTGTGCCC	C17		G17	
TS13-9	GTCAAGCTCAATGCCCC	T14G15T16			
TS13-10	GTCAAGCTCAATGTGACCCC	T16		A16	
TS13-11	GTCAAGCTCAATGTCCCC	G15T16			

TS13-12	GTCAAGCTCAATGTGTTCCC	C17	T17
TS13-13	GTCAAGCTCAATGACCCC	T14G15T16	A14
TS13-14	GTCAAGCTCAATGTGGCCC	T16C17	G16
TS13-15	GTCAAGCTCAATGTGCGCCCC	T16	C16G17
TS13-16	GTCAAGCTCAATGTCCCCC	G15T16	C15
TS13-17	GTCAAGCTCAATGTGGGCCCC	T16	G16G17
TS13-18	GTCAAGCTCAATGTGAGCCC	T16C17	A16G17
TS13-19	GTCAAGCTCAATCCCCC	G13T14G15T16	C13
TS13-20	GTCAAGCTCAATGTGTAACCC	C17	A17A18
TS13-21	GTCAAGCTCAATGTGAGTCCC	T16C17	A16G17T18
TS13-22	GTCAAGCTCAATGTCACCCC	G15T16	C15A16

Table S13B. Off target sites of TS13. Indels are shown in red capital letters. Whereas, PAM sites are shown in blue capital letters.

Line	Target sequence	Deletion	Insertion	Position
OT13-1	ATGTGTCCCCTGG			
OS13-1	ATGTGTACCCTGG	C7	A7	LG2: 6774722-6774734
OS13-2	ATGTGCCCTGG	T6C7		LG2: 6774722-6774734
OS13-3	ATGTGCCCCCTGG	T6	C6	LG2: 6774722-6774734
OS13-4	ATGTCCCCTGG	G5T6		LG2: 6774722-6774734
OS13-5	ATGTAACCCTGG	G5T6C7	A5A6	LG2: 6774722-6774734
OS13-6	ATGTGAACCCTGG	T6C7	A6A7	LG2: 6774722-6774734
OS13-7	ATGTGTTCCCTGG	C7	T7	LG2: 6774722-6774734
OS13-8	ATGTGCCCCTGG	T6		LG2: 6774722-6774734
OS13-9	ATGTACCCCCTGG	G5T6	A5C6	LG2: 6774722-6774734
OS13-10	ATGTGAGCCCTGG	T6C7	A6G7	UGS: 24268-24280
OS13-11	ATGTGGCCCCTGG	T6	G6	UGS: 24268-24280
OS13-12	ATGTGTGCCCTGG	C7	G7	UGS: 24268-24280
OS13-13	ATGTAAGCCCTGG	G5T6C7	A5A6G7	UGS: 24268-24280
OS13-14	ATGTGCCCTGG	T6C7		UGS: 24268-24280
OS13-15	ATGTGTACCCTGG	C7	A7	UGS: 24268-24280
OS13-16	ATGTGACCCCTGG	T6	A6	UGS: 24268-24280
OS13-17	ATGTCACCCCTGG	G5T6	C5A6	UGS: 24268-24280
OS13-18	ATGTACCCTGG	G5T6C7	A5	UGS: 24268-24280
OS13-19	ATGTGTCCCCTGG	T4G5T6		UGS: 24268-24280
OS13-20	ATGTGGACCCTGG	T6C7	G6A7	UGS: 7335-7323
OS13-21	ATGTGTCCCTGG	C7		UGS: 7335-7323
OS13-22	ATGTCCCCTGG	G5T6		UGS: 7335-7323
OS13-23	ATGTGCCCCTGG	T6		UGS: 7335-7323
OS13-24	ATGACACCCCTGG	T4G5T6	A4C5A6	UGS: 7335-7323
OS13-25	ATGTCACCCCTGG	G5T6	C5A6	UGS: 7335-7323
OS13-26	ATGTGAACCCTGG	T6C7	A6A7	UGS: 7335-7323
OS13-27	ATGTGTCCCCTGG	C7	C7	UGS: 7335-7323

OS13-28	ATGTGCCCC**TGG**	T6	UGS: 7335-7323

UGS: unplaced genomic scaffold

Table S14A. Target sequence of TS14 mutant lines. Indels are shown in red capital letters.

Line	Target sequence	Deletion	Insertion
Control	CACCGACGACCCGAAGCAAC		
TS14-1	CACCGACGACCCGAAG**A**AAC	C17	A17
TS14-2	CACCGACGACCCGAACAAC	G16	
TS14-3	CACCGACGACCCGAA**A**AAC	G16C17	A16
TS14-4	CACCGACGACCCGAAGAAC	C17	
TS14-5	CACCGACGACCCGACAAC	A15G16	
TS14-6	CACCGACGACCCGAA**A**CAAC	G16	A16
TS14-7	CACCGACGACCCGA**C**CAAC	A15G16	C15
TS14-8	CACCGACGACCCGAAAAC	G16C17	
TS14-9	CACCGACGACCCGAAG**G**AAC	C17	G17
TS14-10	CACCGACGACCCGCAAC	A14A15G16	
TS14-11	CACCGACGACCCGAA**T**CAAC	G16	T16
TS14-12	CACCGACGACCCGAA**AT**CAAC	G16	A16T17
TS14-13	CACCGACGACCCGA**T**CAAC	A15G16	T15
TS14-14	CACCGACGACCC**C**CAAC	G13A14A15G16	C13
TS14-15	CACCGACGACCCG**C**CAAC	A14A15G16	C14
TS14-16	CACCGACGACCCGAAG**T**AAC	C17	T17
TS14-17	CACCGACGACCCGA**TC**CAAC	A15G16	T15C16
TS14-18	CACCGACGACCC**T**AAC	G13A14A15G16C17	T13
TS14-19	CACCGACGACCCGAA**CA**CAAC	G16	C16A17
TS14-20	CACCGACGACCCGA**CC**CAAC	A15G16	C15C16
TS14-21	CACCGACGACCCGAA**AT**AAC	G16C17	A16T17

Table S14B. Off target sites of TS14. Indels are shown in red capital letters. Whereas, PAM sites are shown in blue capital letters.

Line	Target sequence	Deletion	Insertion	Position
OT14-1	CCGAAGCAAC**GGG**			
OS14-1	CCGAAG**A**AAC**GGG**	C7	A7	LG12: 6758353-6758365
OS14-2	CCGAAAAC**GGG**	G6C7		LG12: 6758353-6758365
OS14-3	CCGAA**C**CAAC**GGG**	G6	C6	LG12: 6758353-6758365
OS14-4	CCGACAAC**GGG**	A5G6		LG12: 6758353-6758365
OS14-5	CCGA**TT**AAC**GGG**	A5G6C7	T5T6	LG12: 6758353-6758365
OS14-6	CCGAAG**G**AAC**GGG**	C7	G7	LG15: 1041360-1041348
OS14-7	CCGAA**AT**AAC**GGG**	G6C7	A6T7	LG15: 1041360-1041348
OS14-8	CCGAAG**T**AAC**GGG**	C7	T7	LG15: 1041360-1041348
OS14-9	CCGA**TAG**AAC**GGG**	A5G6C7	T5A6G7	LG15: 1041360-1041348
OS14-10	CCGAAG**T**AAC**GGG**	C7	T7	UGS: 30232-30244

OS14-11	CCGAATAAACGGG	G6C7	T6A7	UGS: 30232-30244
OS14-12	CCGAAACAACGGG	G6	A6	UGS: 30232-30244
OS14-13	CCGACTCAACGGG	A5G6	C5T6	UGS: 30232-30244
OS14-14	CCGAAGAACGGG	C7		UGS: 30232-30244
OS14-15	CCGAAACGGG	A5G6C7		UGS: 30232-30244
OT14-2	CCGAAGCAACAGG			
OS14-16	CCGAATCAACAGG	G6	T6	LG7: 12504342-12504330
OS14-17	CCGAAAAAACAGG	G6C7	A6A7	LG7: 12504342-12504330
OS14-18	CCGAAGTAACAGG	C7	T7	LG7: 12504342-12504330
OS14-19	CCGATCCAACAGG	A5G6	T5C6	LG7: 12504342-12504330
OS14-20	CCGATCAAACAGG	A5G6C7	T5C6A7	LG16: 535954-535942
OS14-21	CCGAACAACAGG	G6		LG16: 535954-535942
OS14-22	CCGAATAACAGG	G6C7	T6	LG16: 535954-535942
OS14-23	CCGACCCAACAGG	A5G6	C5C6	LG16: 535954-535942
OS14-24	CCGAAGGAACAGG	C7	G7	LG21: 7303144-7303132
OS14-25	CCGATCCAACAGG	A5G6	T5C6	LG21: 7303144-7303132
OS14-26	CCGAAAACAGG	G6C7		LG21: 7303144-7303132
OS14-27	CCGAATCAACAGG	G6	T6	LG7: 12504342-12504330
OS14-28	CCGAATAACAGG	G6C7	T6	LG16: 535954-535942
OS14-29	CCGATCCAACAGG	A5G6	T5C6	LG21: 7303144-7303132

UGS: unplaced genomic scaffold

Table S15A. Target sequence of TS15 mutant lines. Indels are shown in red capital letters.

**Line**	**Target sequence**	**Deletion**	**Insertion**
Control	TCCACGTGCCGTTCACGAGC		
TS15-1	TCCACGTGCCGTTCACCAGC	G17	C17
TS15-2	TCCACGTGCCGTTCAGAGC	C16	
TS15-3	TCCACGTGCCGTTCAAAGC	C16G17	A16
TS15-4	TCCACGTGCCGGAGC	T12T13C14A15C16	
TS15-5	TCCACGTGCCGTTCACAGC	G17	
TS15-6	TCCACGTGCCGTTCAAGC	C16G17	
TS15-7	TCCACGTGCCGTTCGGAGC	A15C16	G15
TS15-8	TCCACGTGCCGTTCGAGC	A15C16	
TS15-9	TCCACGTGCCGTTCATGAGC	C16	T16
TS15-10	TCCACGTGCCGTTCAAGAGC	C16	A16
TS15-11	TCCACGTGCCGTTCACTAGC	G17	T17
TS15-12	TCCACGTGCCGTTCATAGC	C16G17	T16
TS15-13	TCCACGTGCCGTTCTGGAGC	A15C16	T15G16
TS15-14	TCCACGTGCCGTTCAAAGAGC	C16	A16A17
TS15-15	TCCACGTGCCGTTGAGC	C14A15C16	
TS15-16	TCCACGTGCCGTTCACAAGC	G17	A17
TS15-17	TCCACGTGCCGTTCAGGAGC	C16	G16
TS15-18	TCCACGTGCCGTGAGC	T13C14A15C16	

TS15-19	TCCACGTGCCGTTCA**AT**AGC	C16G17	A16T17
TS15-20	TCCACGTGCCG**G**GAGC	T12T13C14A15C16	G12
TS15-21	TCCACGTGCCGTT**G**GAGC	C14A15C16	G14
TS15-22	TCCACGTGCCGTTC**TT**GAGC	A15C16	T15T16

Table S15B. Off target sites of TS15. Indels are shown in red capital letters. Whereas, PAM sites are shown in blue capital letters.

Line	Target sequence	Deletion	Insertion	Position
OT15-1	GTTCACGAGC**GGG**			
OS15-1	GTTCAC**A**AGC**GGG**	G7	A7	LG13: 9975156-9975168
OS15-2	GTTCA**T**GAGC**GGG**	C6	T6	LG13: 9975156-9975168
OS15-3	GTTCAAGC**GGG**	C6G7		LG13: 9975156-9975168
OS15-4	GTTC**TG**GAGC**GGG**	A5C6	T5G6	LG13: 9975156-9975168
OS15-5	GTTCAGC**GGG**	A5C6G7		LG13: 9975156-9975168
OS15-6	GTTCAC**T**AGC**GGG**	G7	T7	UGS: 18298-18310
OS15-7	GTTC**TTA**AGC**GGG**	A5C6G7	T5T6A7	UGS: 18298-18310
OS15-8	GTTCA**G**GAGC**GGG**	C6	G6	UGS: 18298-18310
OS15-9	GTTC**GG**GAGC**GGG**	A5C6	G5G6	UGS: 18298-18310
OS15-10	GTTCAC**C**AGC**GGG**	G7	C7	UGS: 18298-18310
OT15-2	GTTCACGAGC**AGG**			
OS15-11	GTTCA**AT**AGC**AGG**	C6G7	A6T7	LG2: 12534829-12534841
OS15-12	GTTCACAGC**AGG**	G7		LG2: 12534829-12534841
OS15-13	GTTC**GT**GAGC**AGG**	A5C6	G5T6	LG2: 12534829-12534841
OS15-14	GTTCA**A**GAGC**AGG**	C6	A6	LG21: 6539969-6539981
OS15-15	GTTC**TG**GAGC**AGG**	A5C6	T5G6	LG21: 6539969-6539981
OS15-16	GTTCA**AA**AGC**AGG**	C6G7	A6A7	LG24: 2383547-2383535
OS15-17	GTTCAC**C**AGC**AGG**	G7	C7	LG24: 2383547-2383535
OS15-18	GTTCA**G**GAGC**AGG**	C6	G6	LG24: 2383547-2383535
OT15-3	GTTCACGAGC**CGG**			
OS15-19	GTTCAC**A**AGC**CGG**	G7	A7	LG8: 8461595-8461607
OS15-20	GTTCAAGC**CGG**	C6G7		LG8: 8461595-8461607
OS15-21	GTTCA**T**GAGC**CGG**	C6	T6	LG8: 8461595-8461607
OS15-22	GTTC**CT**AGC**CGG**	A5C6G7	C5T6	LG11: 8062556-8062544
OS15-23	GTTC**GT**GAGC**CGG**	A5C6	G5T6	LG11: 8062556-8062544
OS15-24	GTTCAC**A**AGC**CGG**	G7	A7	LG17: 9252828-9252840
OS15-25	GTTCA**TA**AGC**CGG**	C6G7	T6A7	LG17: 9252828-9252840
OS15-26	GTTCA**T**GAGC**CGG**	C6	T6	LG17: 9252828-9252840
OS15-27	GTTCAC**A**AGC**CGG**	G7	A7	LG8: 8461595-8461607
OS15-28	GTTCA**TA**AGC**CGG**	C6G7	T6A7	LG17: 9252828-9252840

UGS: unplaced genomic scaffold

Table S16A. Target sequence of TS16 mutant lines. Indels are shown in red capital letters.

Line	Target sequence	Deletion	Insertion
Control	TCCTGACCCCTATTGTTTAC		
TS16-1	TCCTGACCCCTATTGTATAC	T17	A17
TS16-2	TCCTGACCCCTATTGTTAC	T16	
TS16-3	TCCTGACCCCTATTCTTAC	G15T16	C15
TS16-4	TCCTGACCCCTATTGTTAC	T17	
TS16-5	TCCTGACCCCTATTGCTTAC	T16	C16
TS16-6	TCCTGACCCCTATTGTAC	T16T17	
TS16-7	TCCTGACCCCTATTGTCTAC	T17	C17
TS16-8	TCCTGACCCCTATTAC	T14G15T16T17	
TS16-9	TCCTGACCCCTAATTAC	T13T14G15T16	A13
TS16-10	TCCTGACCCCTATTGATTAC	T16	A16
TS16-11	TCCTGACCCCTATTGTGTAC	T17	G17
TS16-12	TCCTGACCCCTACTTAC	T13T14G15T16	C13
TS16-13	TCCTGACCCCTATTCATTAC	G15T16	C15A16
TS16-14	TCCTGACCCCTATTATTAC	G15T16	A15
TS16-15	TCCTGACCCCTATCTAC	T14G15T16T17	C14
TS16-16	TCCTGACCCCTATTGTAGTAC	T17	A17G18
TS16-17	TCCTGACCCCTATTAC	T13T14G15T16	
TS16-18	TCCTGACCCCTATTGTGAC	T16T17	G16
TS16-19	TCCTGACCCCTATTGACTTAC	T16	A16C17
TS16-20	TCCTGACCCCTATTTTAC	G15T16	
TS16-21	TCCTGACCCCTATTGTGCTAC	T17	G17C18

Table S16B. Off target sites of TS16. Indels are shown in red capital letters. Whereas, PAM sites are shown in blue capital letters.

Line	Target sequence	Deletion	Insertion	Position
OT16-1	TATTGTTTACTGG			
OS16-1	TATTGTTACTGG	T7		LG2: 15954396-15954384
OS16-2	TATTGAGTACTGG	T6T7	A6G7	LG2: 15954396-15954384
OS16-3	TATTGTATACTGG	T7	A7	LG2: 15954396-15954384
OS16-4	TATTTTACTGG	G5T6		LG6: 1247886-1247874
OS16-5	TATTGTGTACTGG	T7	G7	LG6: 1247886-1247874
OS16-6	TATTGACTACTGG	T6T7	A6C7	LG6: 1247886-1247874
OS16-7	TATTGATTACTGG	T6	A6	LG10: 3537581-3537593
OS16-8	TATTGTATACTGG	T7	A7	LG10: 3537581-3537593
OS16-9	TATTGCGTACTGG	T6T7	C6G7	LG10: 3537581-3537593
OS16-10	TATTGCTTACTGG	T6	C6	LG13: 6125105-6125117
OT16-2	TATTGTTTACGGG			
OS16-11	TATTGAATACGGG	T6T7	A6A7	LG1: 1277293-1277305
OS16-12	TATTGTCTACGGG	T7	C7	LG1: 1277293-1277305
OS16-13	TATTACTTACGGG	G5T6	A5C6	LG11: 2199442-2199430
OS16-14	TATTGTTACGGG	T7		LG11: 2199442-2199430

OS16-15	TATTGGTTACGGG	T6	G6	LG11: 2199442-2199430
OS16-16	TATTTTACGGG	G5T6		LG11: 2199442-2199430
OS16-17	TATTGCGTACGGG	T6T7	C6G7	LG17: 150889-150877
OS16-18	TATTGTCTACGGG	T7	C7	LG17: 150889-150877
OT16-3	TATTGTTTACAGG			
OS16-19	TATTGATTACAGG	T6	A6	LG1: 7221465-7221453
OS16-20	TATTGTCTACAGG	T7	C7	LG1: 7221465-7221453
OS16-21	TATTGCATACAGG	T6T7	C6A7	LG1: 7221465-7221453
OS16-22	TATTCATTACAGG	G5T6	C5A6	LG4: 7478227-7478215
OS16-23	TATTGTGTACAGG	T7	G7	LG4: 7478227-7478215
OS16-24	TATTGAATACAGG	T6T7	A6A7	LG4: 7478227-7478215
OT16-4	TATTGTTTACCGG			
OS16-25	TATTGTCTACCGG	T7	C7	LG6: 10746663-10746675
OS16-26	TATTGCTTACCGG	T6T7	C6T7	LG6: 10746663-10746675
OS16-27	TATTGCTTACCGG	T6	C6	LG6: 10746663-10746675
OS16-28	TATTGTATACCGG	T7	A7	LG8: 6221221-6221233
OS16-29	TATTGCGTACCGG	T6T7	C6G7	LG8: 6221221-6221233

Table S17A. Target sequence of TS17 mutant lines. Indels are shown in red capital letters.

Line	Target sequence	Deletion	Insertion
Control	ATCTTCTTTGCTTTCTCTTA		
TS17-1	ATCTTCTTTGCTTTCTATTA	C17	A17
TS17-2	ATCTTCTTTGCTTTCCTTA	T16	
TS17-3	ATCTTCTTTGCTTTCATTA	T16C17	A16
TS17-4	ATCTTCTTTGCTTTTTA	C15T16C17	
TS17-5	ATCTTCTTTGCTTTCTTTA	C17	
TS17-6	ATCTTCTTTGCTTGCTTA	T14C15T16	G14
TS17-7	ATCTTCTTTGCTTTCCCTTA	T16	C16
TS17-8	ATCTTCTTTGCTATTA	T13T14C15T16C17	A13
TS17-9	ATCTTCTTTGCTTTCTTA	T16C17	
TS17-10	ATCTTCTTTGCTTTCTTTTA	C17	T17
TS17-11	ATCTTCTTTGCTCTTA	T13T14C15T16	
TS17-12	ATCTTCTTTGCTTTCTACTTA	T16	A16
TS17-13	ATCTTCTTTGCTTTCGATTA	T16C17	G16A17
TS17-14	ATCTTCTTTGCTTTCTGTTA	C17	G17
TS17-15	ATCTTCTTTGCTTTCCCCTTA	T16	C16C17
TS17-16	ATCTTCTTTGCTTCTTA	T14C15T16	
TS17-17	ATCTTCTTTGCTTTATTA	C15T16C17	A15
TS17-18	ATCTTCTTTGCTTTCGGTTA	T16C17	G16G17
TS17-19	ATCTTCTTTGCTGCTTA	T13T14C15T16	G13
TS17-20	ATCTTCTTTGCTTTCTAGTTA	C17	A17G18
TS17-21	ATCTTCTTTGCTTTCGACTTA	T16	G16A17

Table S17B. Off target sites of TS17. Indels are shown in red capital letters. Whereas, PAM sites are shown in blue capital letters.

Line	Target sequence	Deletion	Insertion	Position
OT17-1	CTTTCTCTTA**TGG**			
OS17-1	CTTTCT**A**TTA**TGG**	C7	A7	LG4: 3362530-3362518
OS17-2	CTTTC**GA**TTA**TGG**	T6C7	G6A7	LG4: 3362530-3362518
OS17-3	CTTTC**A**CTTA**TGG**	T6	A6	LG4: 3362530-3362518
OS17-4	CTTT**AG**CTTA**TGG**	C5T6	A5G6	LG4: 3362530-3362518
OS17-5	CTTTCT**T**TTA**TGG**	C7	T7	LG12: 979999-979987
OS17-6	CTTTTTA**TGG**	C5T6C7		LG12: 979999-979987
OS17-7	CTTTC**AA**TTA**TGG**	T6C7	A6A7	LG12: 979999-979987
OS17-8	CTTTC**C**CTTA**TGG**	T6	C6	LG21: 6645647-6645635
OS17-9	CTTTCT**G**TTA**TGG**	C7	G7	LG21: 6645647-6645635
OS17-10	CTTT**GG**CTTA**TGG**	C5T6	G5G6	LG21: 6645647-6645635
OT17-2	CTTTCTCTTA**AGG**			LG21: 6645647-6645635
OS17-11	CTTTCT**A**TTA**AGG**	C7	A7	LG1: 9465340-9465328
OS17-12	CTTT**GAA**TTA**AGG**	C5T6C7	G5A6A7	LG1: 9465340-9465328
OS17-13	CTTTC**G**CTTA**AGG**	T6	G6	LG1: 9465340-9465328
OS17-14	CTTTC**GG**TTA**AGG**	T6C7	G6G7	LG6: 4623081-4623093
OS17-15	CTTTCT**T**TTA**AGG**	C7	T7	LG6: 4623081-4623093
OS17-16	CTTTCTTA**AGG**	C5T6		LG6: 4623081-4623093
OS17-17	CTTT**AG**CTTA**AGG**	C5T6	A5G6	LG8: 4813250-4813238
OS17-18	CTTTC**A**CTTA**AGG**	T6	A6	LG8: 4813250-4813238
OT17-3	CTTTCTCTTA**CGG**			LG8: 4813250-4813238
OS17-19	CTTTCTTTA**CGG**	C7		LG9: 12609868-12609856
OS17-20	CTTT**AA**CTTA**CGG**	C5T6	A5A6	LG9: 12609868-12609856
OS17-21	CTTTC**AG**TTA**CGG**	T6C7	A6G7	LG9: 12609868-12609856
OS17-22	CTTTC**C**CTTA**CGG**	T6	C6	LG16: 7340866-7340878
OS17-23	CTTT**GGA**TTA**CGG**	C5T6C7	G5G6A7	LG16: 7340866-7340878
OS17-24	CTTT**GA**CTTA**CGG**	C5T6	G5A6	LG16: 7340866-7340878
OS17-25	CTTTCT**A**TTA**CGG**	C7	A7	LG16: 7340866-7340878
OS17-26	CTTTCTTA**CGG**	T6C7		LG25: 2895215-2895227
OS17-27	CTTTC**A**CTTA**CGG**	T6	A6	LG25: 2895215-2895227
OS17-28	CTTTC**C**CTTA**CGG**	T6	C6	LG16: 7340866-7340878
OS17-29	CTTTCT**A**TTA**CGG**	C7	A7	LG16: 7340866-7340878

Table S18A. Target sequence of TS18 mutant lines. Indels are shown in red capital letters.

Line	Target sequence	Deletion	Insertion
Control	CGATCCGAATTCGCCGAAAG		
TS18-1	CGATCCGAATTCGCCGAAG	A17	
TS18-2	CGATCCGAATTCGCC**A**AAAG	G16	A16
TS18-3	CGATCCGAATTCGCCAAG	G16A17	
TS18-4	CGATCCGAATTCGC**T**AAG	C15G16A17	T15

TS18-5	CGATCCGAATTCGAAAG	C14C15G16	
TS18-6	CGATCCGAATTCGCCAAAG	G16	
TS18-7	CGATCCGAATTCGCCGCAAG	A17	C17
TS18-8	CGATCCGAATTCGCCCAAG	G16A17	C16
TS18-9	CGATCCGAATTCGTAAG	G13C14C15G16A17	T13
TS18-10	CGATCCGAATTCGCAAG	C15G16A17	
TS18-11	CGATCCGAATTCGCCTAAAG	G16	T16
TS18-12	CGATCCGAATTCGCCTAAG	G16A17	T16
TS18-13	CGATCCGAATTCGCCGGAAG	A17	G17
TS18-14	CGATCCGAATTCGAAAAG	C14C15G16	A14
TS18-15	CGATCCGAATTCAAAG	G13C14C15G16	
TS18-16	CGATCCGAATTCGCCTAAAAG	G16	T16A17
TS18-17	CGATCCGAATTCGCCTCAAG	G16A17	T16C17
TS18-18	CGATCCGAATTCGCCGTCAAG	A17	T17C18
TS18-19	CGATCCGAATTCAAAAG	G13C14C15G16	A13
TS18-20	CGATCCGAATTCGCCTTAAG	G16A17	T16T17
TS18-21	CGATCCGAATTCGCCCCAAAG	G16	C16C17
TS18-22	CGATCCGAATTCGCCGTAAG	A17	T17

Table S18B. Off target sites of TS18. Indels are shown in red capital letters. Whereas, PAM sites are shown in blue capital letters.

Line	Target sequence	Deletion	Insertion	Position
OT18-1	TCGCCGAAAG**GGG**			
OS18-1	TCGCCGTAAG**GGG**	A7	T7	LG5: 10382051-10382063
OS18-2	TCGCCCAAAG**GGG**	G6	C6	LG5: 10382051-10382063
OS18-3	TCGCCAAG**GGG**	G6A7		LG5: 10382051-10382063
OS18-4	TCGCCGCAAG**GGG**	A7	C7	LG5: 10382051-10382063
OS18-5	TCGCTAAAAG**GGG**	C5G6	T5A6	LG5: 10382051-10382063
OS18-6	TCGCAAG**GGG**	C5G6A7		LG5: 10382051-10382063
OS18-7	TCGCCAAAAG**GGG**	G6	A6	LG2: 11993964-11993952
OS18-8	TCGCCCTAAG**GGG**	G6A7	C6T7	LG2: 11993964-11993952
OS18-9	TCGCAAAAAG**GGG**	C5G6	A5A6	LG2: 11993964-11993952
OS18-10	TCGCCGGAAG**GGG**	A7	G7	LG2: 11993964-11993952
OT18-2	TCGCCGAAAG**AGG**			
OS18-11	TCGCCTAAAG**AGG**	G6	T6	LG1: 13530845-13530857
OS18-12	TCGCCGTAAG**AGG**	A7	T7	LG1: 13530845-13530857
OS18-13	TCGCCAAG**AGG**	G6A7		LG1: 13530845-13530857
OS18-14	TCGCTTCAAG**AGG**	C5G6A7	T5T6C7	LG2: 5664082-5664070
OS18-15	TCGCAAAG**AGG**	C5G6		LG2: 5664082-5664070
OS18-16	TCGCCGCAAG**AGG**	A7	C7	LG2: 5664082-5664070
OS18-17	TCGCCAAAG**AGG**	G6		LG5: 10929508-10929496
OS18-18	TCGCATAAAG**AGG**	C5G6	A5T6	LG5: 10929508-10929496
OT18-3	TCGCCGAAAG**TGG**			

OS18-19	TCGCC**C**AAAG**TGG**	G6	C6	LG2: 7692275-7692263
OS18-20	TCGCCG**G**AAG**TGG**	A7	G7	LG2: 7692275-7692263
OS18-21	TCGC**TAC**AAG**TGG**	C5G6A7	T5A6C7	LG2: 7692275-7692263
OS18-22	TCGCC**TT**AAG**TGG**	G6A7	T6T7	LG5: 8447682-8447694
OS18-23	TCGCAAAG**TGG**	C5G6		LG5: 8447682-8447694
OS18-24	TCGCC**T**AAAG**TGG**	G6	T6	LG5: 8447682-8447694
OS18-25	TCGCCG**G**AAG**TGG**	A7	G7	LG5: 8447682-8447694
OS18-26	TCGCC**CT**AAG**TGG**	G6A7	C6T7	LG12: 9301338-9301350
OS18-27	TCGCCG**G**AAG**TGG**	A7	G7	LG2: 7692275-7692263
OS18-28	TCGCCG**G**AAG**TGG**	A7	G7	LG5: 8447682-8447694
